# Restricting epigenetic activity promotes the reprogramming of transformed cells to pluripotency in a line-specific manner

**DOI:** 10.1038/s41420-023-01533-8

**Published:** 2023-07-14

**Authors:** Xiuling Fu, Qiang Zhuang, Isaac A. Babarinde, Liyang Shi, Gang Ma, Haoqing Hu, Yuhao Li, Jiao Chen, Zhen Xiao, Boping Deng, Li Sun, Ralf Jauch, Andrew P. Hutchins

**Affiliations:** 1 Department of Systems Biology, School of Life Sciences, Southern University of Science and Technology, Shenzhen, 518055 China; 2 School of Biomedical Sciences, Li Ka Shing Faculty of Medicine, The University of Hong Kong, Hong Kong SAR, China; 3 Centre for Translational Stem Cell Biology, Hong Kong SAR, China

**Keywords:** Reprogramming, Cancer stem cells, Differentiation

## Abstract

Somatic cell reprogramming and oncogenic transformation share surprisingly similar features, yet transformed cells are resistant to reprogramming. Epigenetic barriers must block transformed cells from reprogramming, but the nature of those barriers is unclear. In this study, we generated a systematic panel of transformed mouse embryonic fibroblasts (MEFs) using oncogenic transgenes and discovered transformed cell lines compatible with reprogramming when transfected with *Oct4*/*Sox2*/*Klf4*/*Myc*. By comparing the reprogramming-capable and incapable transformed lines we identified multiple stages of failure in the reprogramming process. Some transformed lines failed at an early stage, whilst other lines seemed to progress through a conventional reprogramming process. Finally, we show that MEK inhibition overcomes one critical reprogramming barrier by indirectly suppressing a hyperacetylated active epigenetic state. This study reveals that diverse epigenetic barriers underly resistance to reprogramming of transformed cells.

## Introduction

Transformed cells and embryonic stem cells (ESCs) have a remarkable list of similarities [1]. Both cell types have a relaxed chromatin state [2], adopt a glycolysis-biased metabolism despite the availability of oxygen [3, 4], can undergo an epithelial-mesenchymal transition (EMT) [5], and form teratomas [6]. Transformed cells acquire features reminiscent of embryonic development, such as increased cellular plasticity and the upregulated expression of pluripotent genes, including *OCT4*, *NANOG*, and *SOX2* [7]. Indeed, the expression of pluripotent-specific genes in patient tumor samples correlates with poor clinical outcomes [8, 9]. For example, OCT4 expression is associated with germ cell tumors and cancer stem cells [10, 11], SOX2 expression with glioblastoma [12], and *NANOG* with colorectal [13] and prostate cancer [14].

Somatic cells can be reprogrammed into induced pluripotent stem cells (iPSCs) by the transfection of a cocktail of transgenes, particularly *Oct4* (*Pou5f1*), *Sox2*, *Klf4*, and *Myc* [15]. Curiously, despite being an artificial process, the reprogramming of somatic cells to iPSCs passes through distinct phases, reminiscent of a developmental program [16–19]. Tumorigenic transformation also passes through a series of distinct phases in a process that has similarities to somatic cell reprogramming [1, 20]. Some studies suggest direct links between reprogramming and cancer. For example, transient in vivo activation of reprogramming factors leads to tumor formation [21, 22], and cancer-associated mutations in the transcription factor *SOX17* can confer reprogramming capability to the normally incapable SOX17 [23].

There have been reports on reprogramming primary human cancer cells to an embryonic state, including cancerous cells from the liver, gastrointestinal tract, and sarcoma cell lines [24–27]. However, the bona fide reprogramming of these lines is not always clear. Blood cancer cells seem more amenable: human KBM7 cells, T cell acute lymphoblastic leukemia, acute myeloid leukemia [28–31], lymphoblastoid cells [32, 33], and chronic myeloid leukemia cells [27, 34–36] all have reports of successful reprogramming. However, the process of reprogramming is inefficient and often incomplete, and transformed cells are resistant to reprogramming [31, 37]. Additionally, it is unclear how closely the iPSC-like cells resemble iPSCs. For example, the overall gene expression of the reprogrammed cancer cells remains distinct from genuine ESCs/iPSCs [26, 32, 38]. A significant problem is the reprogramming of untransformed bystander cells from primary cancer tissues [31, 39]. Finally, the efficiency of reprogramming transformed cells is much lower than wildtype reprogramming. This is a curious contradiction. Considering the similarities between cancer cells and iPSCs and the pathways used to generate them, it seems that reprogramming should be easier in transformed cells as they have reduced barriers to cell-type conversion. A deeper understanding of the relationship between reprogramming and tumorigenic transformation may shed light on mechanisms of cell type control in cancer development. Additionally, reprogramming transformed cells to a normal iPSC-like state can model cancer development as tumorigenesis can be studied not only in the tissue type where the tumor originated but also in other cell types [29, 31]. These models could be used to explore the earliest stages of cancer development that are usually hidden in humans.

To explore the epigenetic barriers blocking the reprogramming of transformed cells, we generated a panel of ten artificial transformed mouse cell lines. Seven of these lines can acquire OCT4-GFP+ reporter expression and pluripotent characteristics using a conventional OSKM-reprogramming protocol, albeit at very low efficiency. Of the remaining three reprogramming-incapable lines, we show that the defects in reprogramming are line-specific. Reprogramming is a phased process and some lines fail in the early phases, others in the later phases. Compared to wildtype cells, the reprogramming-incapable lines show a heightened ‘hyperactive’ chromatin state and demonstrate global increases in chromatin accessibility and histone acetylation. Some of the transformed lines could be converted to reprogramming-capable by inhibiting MEK signaling which moderates the active chromatin state and leads to decreased histone acetylation.

## Results

### Transformed mouse cell lines are resistant to reprogramming

**Fig. 1:**
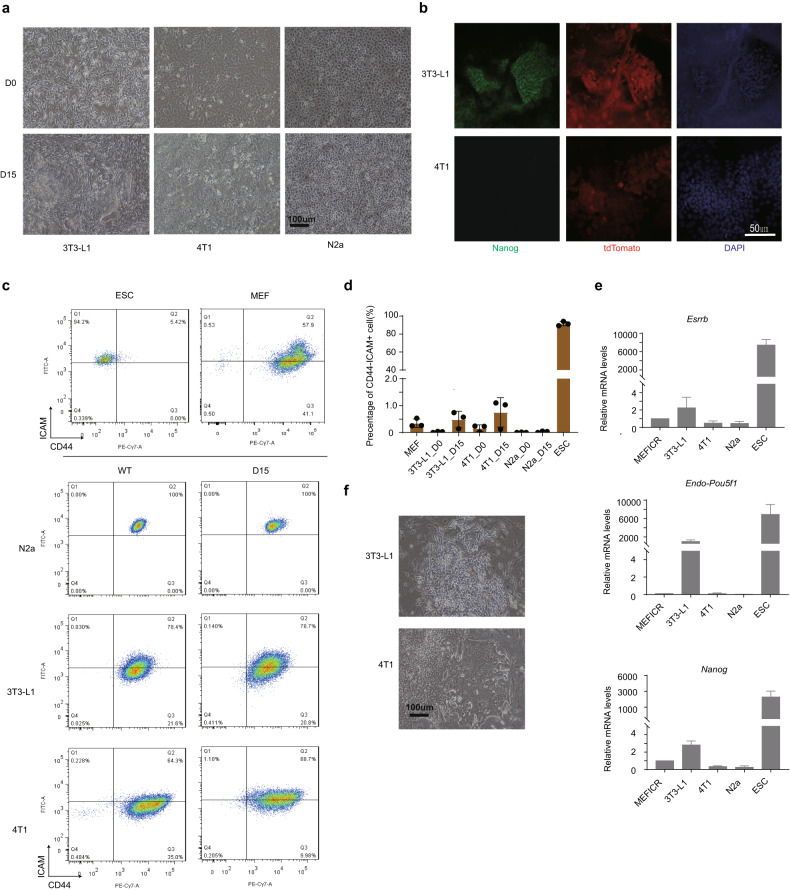
Transformed cell lines are highly resistant to reprogramming. **a** Bright-field images of the reprogramming of 3T3-L1, 4T1, and N2a cell lines in serum+Vc reprogramming conditions. Images are shown from day 0 (D0) or day 15 (D15) of the reprogramming experiment. Morphologically iPSC-like cells can typically be observed at day 15 and onwards in wildtype reprogramming. Scale bar = 100 µm. **b** Fluorescent microscopy images for NANOG and tdTomato expressed from the OSKM transgene cassette for the reprogramming of 3T3-L1 and 4T1 cells, from two biological replicates with three images per slide. Scale bar = 50 µm. **c** Example FACS (fluorescent activated cell sorting) plots for wildtype MEFs and mESCs and cells from a reprogramming time course at day 0 or 15 from 3T3-L1, 4T1, and N2a cells. **d** Bar chart of the percentage of ICAM+/CD44− sorted cells in the indicated cell lines at day 0 or day 15 of reprogramming. Data is from the mean of three biological replicates. **e** RT-qPCR showed the indicated gene expression from pooled cultures of the sorted ICAM+ and CD44− cells from the indicated cell lines five days after replating. The mean of four biological replicates, each with three technical replicates ± s.d. is shown. **f** Bright-field images of the sorted ICAM+ and CD44− cells from the indicated cell lines five days after replating. Scale bar = 100 µm.

To explore reprogramming in transformed cell lines we first reprogramed established mouse cell lines that were either spontaneously transformed or derived from a primary tumor. We chose three mouse cell lines: 3T3-L1, a spontaneously immortalized fibroblastic cell line; 4T1, a metastatic breast tumor cell line; and N2a (Neuro-2a), a neuroblastoma cell line (Fig. 1a). We attempted to reprogram these cell lines using OSKM in serum+Vc (Vitamin C). Vc was added to all reprogramming conditions, unless otherwise indicated, to accelerate reprogramming [40]. The 3T3-L1 and 4T1 (but not the N2a) cells formed colonies that morphologically resembled iPSCs on day 15 (Fig. 1a). We did not attempt to reprogram for longer than 15 days as the transformed cell lines grow rapidly and outcompeted the morphological iPSC-like colonies. Low levels of NANOG protein could be detected by immunofluorescence in some 3T3-L1 colonies, but not in 4T1 cells (Fig. 1b). We manually picked the iPSC-like colonies to establish iPSC lines. However, contaminating non-reprogrammed 3T3-L1 and 4T1 cells would outcompete iPSC-like colonies, and based on morphology, the cultures would revert to homogenous 3T3-L1 or 4T1 cultures within one passage. To select for reprogramming cells we used a system involving ICAM and CD44 staining as ICAM+/CD44- cells correlate with the expression of NANOG [41]. We detected small numbers of ICAM+/CD44- cells (~1–1.5%) at day 15 of reprogramming in 3T3-L1 and 4T1, but not N2a cells (Fig. 1c, d and Supplementary Table 1 for this and subsequent figures). qRT-PCR of the ICAM+/CD44- cells indicated that *Esrrb* and *Nanog* were upregulated in the 4T1, and *Essrb*, *Nanog*, and endogenous-*Pou5f1* in 3T3-L1 (Fig. 1e). However, when the sorted cells were replated, they rapidly reverted to the original cell type morphology and there was no evidence of iPSC-like cells (Fig. 1f). Whilst there was some evidence of NANOG protein in the 3T3-L1 cells, there was no expression of NANOG in the 4T1, despite some cells being ICAM+/CD44− (Fig. 1b–d). ICAM+/CD44− cells correlate closely with NANOG expression, however, there was not a perfect match in the original study, and some cells remained ICAM+/CD44−/NANOG- [41]. Our reprogrammed cells are likely failing to commit to a pluripotent state. Potentially, reprogramming for longer than 15 days may allow the derivation of iPSC-like cells if appropriate cell culture and sorting conditions could be designed to select reprogrammed cells.

### Untransformed MEFs senesce and fail to reprogram

Wildtype MEFs can only reprogram at early passages [42], and efficiency declines rapidly before complete failure after passage 4 (Fig. S1a) [43]. RNA-seq of MEFs from passages 1-6 revealed changes in cell cycle genes, including the downregulation of cyclins and other positive cell cycle regulators and the activation of cell cycle inhibitors, particularly *Cdkn1a* (Fig. S1b). We divided the samples into MEFs that could reprogram successfully (P1, P2) or failed (P5, P6) and measured significantly differentially expressed (DE) genes. In total, 337 genes were significantly upregulated and 419 significantly downregulated (Fig. S1c, d). Gene ontology (GO) and gene set enrichment analysis (GSEA) of the differentially expressed genes indicated that the downregulated genes were related to the cell cycle and extracellular matrix. Upregulated genes were related to cell migration/adhesion, MAPK activity, apoptosis, inflammation, and chemokine expression (Fig. S1e–g). We defined the upregulated gene set as the ‘MEF senescent signature’ and the downregulated gene set as a ‘reprogramming permissive signature’ (Supplementary Table S2).

### Generation of a panel of oncogenic transformed MEF cell lines

As the transformed cell lines have been maintained in culture for an extended period, they have accumulated genetic alterations to adapt to the cell culture environment. These changes may permanently impair their ability to reprogram. Consequently, to achieve a controlled system, we generated immortalized MEFs using 10 different combinations of factors to represent a spectrum of transformed cells. The factors were chosen to cover different methods of immortalization: oncogenic transcription factors (Myc, Hras^G12V^, Mef2d, p53DD), viral transforming factors (SV40T, E1A), anti-apoptotic factors (Bcl2), and an engineered epigenetic factor (Hdac7SA; serines at positions 178, 344, and 479 substituted with alanine, to block nuclear export) [44–46] (Supplementary Table S3). Not all of these factors could transform MEFs, and, except for SV40T, at least two factors were required for successful transformation (Fig. S2a, b). The immortalized cell lines were maintained for at least 1 month to remove any non-transformed background MEFs, and the continued expression of transgenes was confirmed by RNA-seq, RT-qPCR, and western blot (Fig. S2c–e). The exception was the p53DD.Myc lines, where Myc could be detected, but p53DD protein could not (Fig. S2c–e). This suggests that both transgenes are required to generate immortal MEFs, but once immortalized, p53DD becomes dispensable and Myc is sufficient.

We next looked at the features of the transformed cells. All of the immortalized MEFs proliferated faster than the wildtype MEFs (Fig. S2f). Hras.E1A, SV40T, and Hras.Myc could form tumors when injected into nude (BALB/cNj-Foxn1^nu^/Gpt) mice, whilst Bcl2.Myc and Hdac7SA.Mef2d could not (Fig. S2g, h). Hematoxylin and eosin staining of cross-sections through the tumors indicated various differentiation layers, although mainly mesoderm (Fig. S2i). Aneuploidy is a common feature of cancer, although its role in transformation remains unclear [47]. A normal karyotype is required for post-implantation embryonic development [48] but may be compatible with pluripotency [49–51]. Nonetheless, to rule out the impact of karyotype abnormalities on reprogramming capability we confirmed a normal karyotype for four of the transformed MEF lines that the study will mainly focus on (Fig. S2j).

### Transformed cell lines have a spectrum of reprogramming capability

**Fig. 2:**
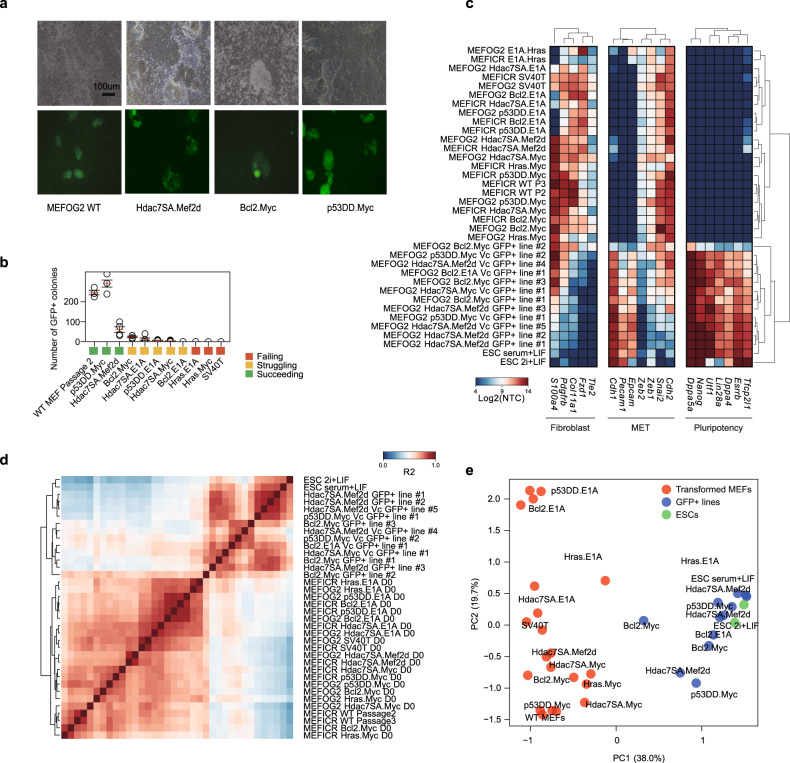
Characterization of transformed fibroblast cell lines capable and incapable of reprogramming to iPSC-like cells. **a** Representative brightfield and GFP images from reprogrammed transformed MEFs for the indicated cell lines. Scale bar = 100 µm. **b** Chart showing the number of Oct4-GFP+ colonies from the indicated transformed MEF lines. Each MEF line was at least 1 month old. Reprogramming was performed between three and five independent biological replicates (See Supplementary Table 1). Circles represent biological replicates; the red line indicates the mean and the error bars indicate the standard error of the mean (SEM). **c** Heatmap from the RNA-seq data showing the expression of a representative set of genes specific to somatic fibroblastic cells, pluripotent stem cells, or from the MET (mesenchymal-epithelial transition). NTC=normalized tag count. **d** Correlation heatmap (R^2^) of the original MEF samples and the reprogrammed GFP+ cells and ESCs. **e** PCA scatter plot showing the first two dimensions of the RNA-seq data from the original wildtype or transformed MEF lines (red) and the GFP+ cell lines (blue) and ESCs (green) maintained in serum+LIF or 2i+LIF.

The immortalized MEF cell lines were reprogrammed using a polycistronic lentiviral OSKM system with vitamin C (Vc), to promote reprogramming [43]. We used transformed lines derived from OG2 MEFs, which contain an Oct4-GFP reporter [43]. Surprisingly, several lines generated GFP+ colonies. Based upon the number of GFP+ colonies generated, we labeled the lines as ‘succeeding’ (Hdac7SA.Mef2d, p53DD.Myc), ‘struggling’ (Bcl2.Myc, Hdac7SA.E1A, p53DD.E1A, Hdac7SA.Myc), and ‘failing’ (Bcl2.E1A, Hras.E1A, Hras.Myc, and SV40T) (Fig. 2a, b). Reprogramming the transformed lines was less efficient than wildtype MEFs, except for the p53DD.Myc line. Complete reprogramming requires passages before the pluripotency gene expression program can be stably established. This process partially relies on the fast-dividing iPSCs outcompeting the slow-growing/senescent wild-type MEFs, however, the transformed MEFs were also highly proliferative (Fig. S2f) and would compete with the reprogrammed iPSCs. Hence, we FACS sorted the GFP+ cells and passaged them to establish iPSC lines in the absence of transformed MEFs. In a panel of marker genes (Fig. 2c), the expression of fibroblastic genes was surprisingly noisy in the transformed MEFs, suggesting transformation has a strong effect. The transformed MEFs retained high expression of mesenchymal genes (*Cdh2*, *Snai2*, *Zeb1*, and *Zeb2*) and low epithelial genes (*Epcam*, *Pecam1*, and *Cdh1*), whilst the reprogrammed iPSC-like cells had the opposite pattern (Fig. 2c). The expression of the pluripotency markers was validated in the iPSC-like lines by immunofluorescence staining (NANOG), RT-qPCR (*Esrrb*, endogenous-*Pou5f1*, *Nanog*), and Western blot (SOX2 and NANOG) (Fig. S3a–c).

The reprogrammed iPSC-like lines had high levels of pluripotency genes and low levels of somatic genes (Fig. 2c), and cross-correlation and principal component analysis (PCA) indicated the cells were similar to ESCs (Fig. 2d, e). We used DPre, a computational tool that identifies the cell type based on RNA-seq expression [52, 53]. DPre identified most GFP+ lines as ESC-like (Fig. S3d, e). The exception was Bcl2.Myc GFP+ Line #2, which had only a weak ESC-like character (Fig. S3d). Additionally, DPre also indicated that several GFP+ lines (Hdac7SA.Mef2d GFP+ line #4, Bcl2.E1A Vc GFP+ line #1, Bcl2.Myc GFP+ line #3) were contaminated with transformed MEFs (Fig. S3d, e). Normal reprogramming relies upon untransformed MEFs senescing and being outcompeted by rapidly growing iPSCs. However, the transformed MEFs can grow as fast as iPSCs and no longer senesce, meaning contamination of iPSC cultures remains a problem.

We confirmed that the iPSC lines could form teratomas with tissues representing all three germ layers (Fig. S3f). A mark of complete reprogramming is the silencing of the OSKM transgenes, and the OSKM transgene was not detected in RNA-seq data (Fig. S3g). Interestingly, the immortalization factors were only partially silenced. All were silenced except E1A in the Bcl2.E1A GFP+ line and Myc in the Bcl2.Myc GFP+ lines (Fig. S3g). We confirmed Hdac7SA and Mef2d were silenced by qRT-PCR (Fig. S3h). Overall, these data confirmed the successful generation of iPSC-like lines from some transformed tumorigenic MEFs.

### Transformed MEFs have multiple transcriptional perturbations

We next explored the properties that made some immortal MEFs reprogramming-capable and others –incapable. We focused on two (non-exclusive) models: the inability to reprogram occurs due to problems in the originating MEFs, or it is caused by failures to traverse the correct sequence of events required for successful reprogramming.

**Fig. 3:**
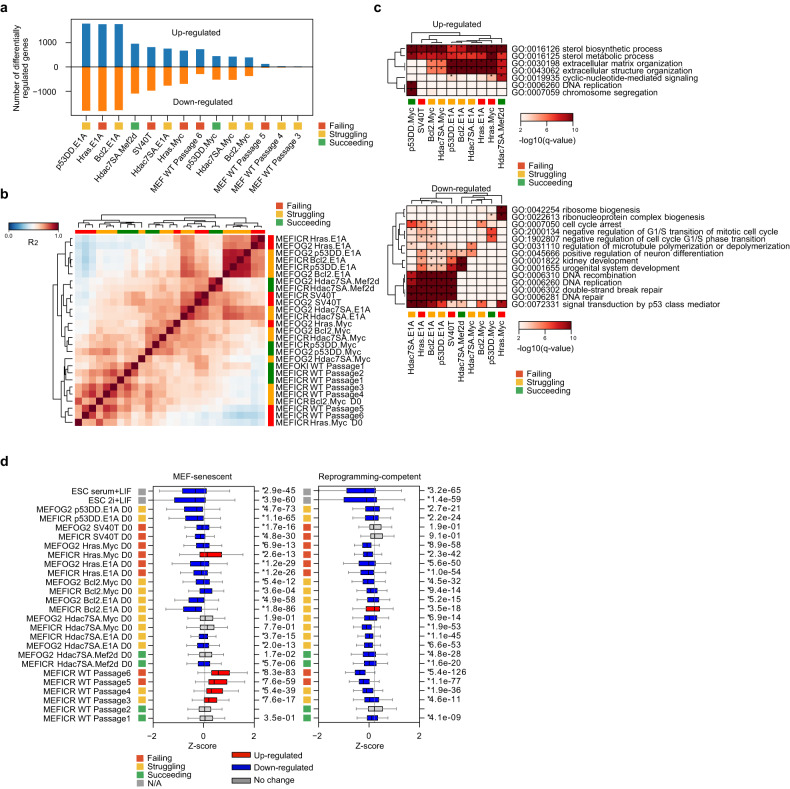
The immortalized MEFs are widely divergent from wildtype MEFs. **a** Chart showing the number of significantly differentially regulated genes up (+) or down (−) regulated (Bonferroni-Hochberg corrected *p*-value of <0.01 and a minimum fold-change of 2). **b** Correlation (R^2^) of the immortalized MEF lines compared to the wildtype MEFs. **c** Selected GO terms from the significantly up or downregulated genes from the transformed MEF lines. A term was considered significant if it had a Bonferroni-Hochberg corrected p-value of <0.01). See also Fig. S4b for the full table. **d** Boxplots for the genes defined as the MEF-senescent signature or the reprogramming-capable signature from Fig. S1c. Significance is from a two-sided Welch’s *t*-test for pair-wise comparisons versus MEFICR wildtype Passage 2 as the reference. A change was considered significant if the *p*-value was <0.01. Significant changes are colored red or blue.

Immortalization of somatic adult cells occurs by a range of mechanisms and is accompanied by changes in the epigenetic state and gene expression patterns. The RNA-seq data of the transformed MEFs showed substantial changes in gene expression and a large variation versus wildtype MEFs (Figs. 2c, e, and 3a). There was no simple correlation between the number of gene changes and reprogramming capability (Fig. 3a). Despite a large number of gene expression changes, there was no evidence that the MEFs were transdifferentiating as their global gene expression patterns continued to correlate well against wildtype MEFs (Fig. 3b), and DPre continued to identify the cells as MEF-like (Fig. S4a). Gene ontology of the DE genes suggested changes were associated with basic cellular processes such as upregulation of metabolic processes, extracellular matrix genes, and downregulation of ribosomes, cell cycle, and DNA repair processes (Fig. 3c, S4b). The MEF-senescent gene signature (defined in Fig. S1c) was not upregulated in the transformed lines, and the majority of lines had a significantly reduced MEF-senescent signature, reminiscent of the level in ESCs (Fig. 3d). This indicates the transformed lines are avoiding senescence. Interestingly, the reprogramming-competent signature (defined in Fig. S1c) declines in all lines (except SV40T), which suggests a reduced capability to reprogram (Fig. 3d).

Surprisingly, the patterns of the gene expression changes were uniform across cell lines; genes up or downregulated in one transformed line tended to be either unchanged or similarly deregulated in other lines (Fig. S4c). This implies a shared transformation signature.

We next looked at specific genes identified as key regulators of the reprogramming process. Three factors related to cancer and cellular transformation that modulate reprogramming in wildtype cells are *Tp53*, *Cdkn1a*, and *Rb1* (Retinoblastoma) [45, 54–57]. There was little change in *Rb1* mRNA levels, and whilst *Cdkn1a* was relatively high in all cell lines except p53DD.E1A, SV40T, and wildtype MEFs, its expression was not correlated with reprogramming capability (Fig. S5a). For *Tp53*, its expression was relatively consistent across the MEF lines and did not correlate with reprogramming ability (Fig. S5a). We inferred p53 activity by looking at known target genes of p53, and all lines showed unaltered p53 activity except for the MEF line containing the dominant-negative p53DD (p53DD.E1A). This line had significantly downregulated p53 target genes (Fig. S5b–c), reduced p53 phosphorylation, and reduced MDM2 protein levels (Fig. S5d). Loss of p53 is beneficial for reprogramming in wildtype cells [45, 57, 58], yet in transformed lines reduced p53 activity in the p53DD.E1A line did not correlate with efficient reprogramming (Fig. 2b). This suggests that, in contrast to wildtype cells, reduced p53 activity is not beneficial for reprogramming in transformed cells.

### Transformed MEFs encounter multiple roadblocks at different phases of reprogramming

**Fig. 4:**
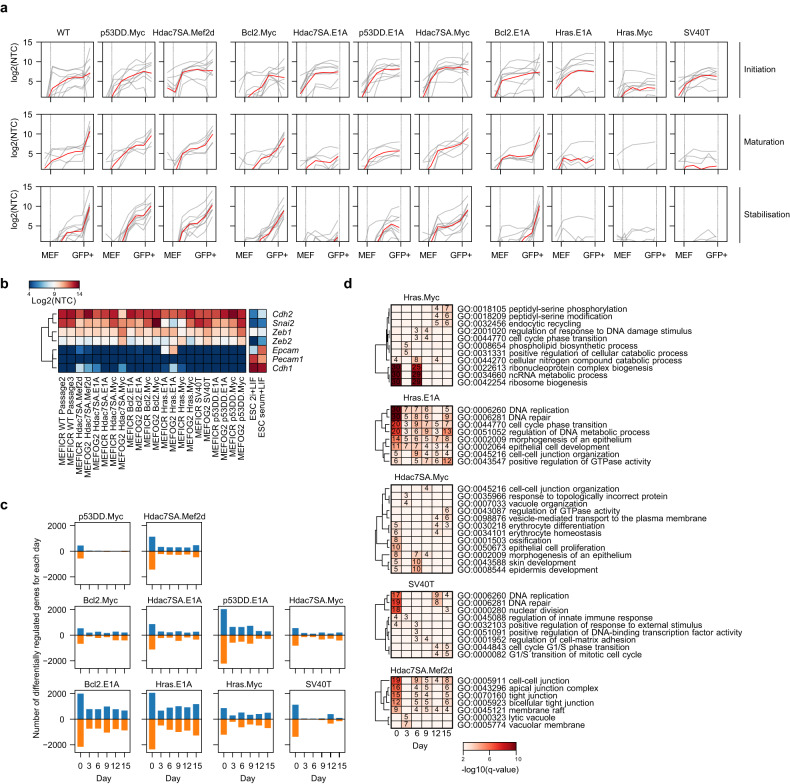
Transformed MEFs fail at different phases of reprogramming. **a** Line charts showing the expression of initiation, maturation, and stabilization genes [59], at the indicated time points or in MEFs and GFP+ cells. See Fig. S6 for the expression of the individual genes in each class. The red line is the mean of all genes, the grey lines are the individual genes. **b** Heatmap of the expression of mesenchymal and epithelial marker genes in starting lines and ESCs. **c** Bar charts showing the number of significantly differentially regulated genes at each time point for the indicated transformed lines versus a wildtype reprogramming time course. Differential expression was compared for each line and time point versus the corresponding wildtype timepoint. A gene was considered differentially regulated if the *q*-value (Bonferroni-Hochberg corrected *p*-value) was <0.01 and the fold-change >2. **d** Heatmaps of significantly overrepresented gene ontology (biological process) terms for downregulated genes from the indicated time points and cell lines.

Reprogramming occurs in defined phases [16, 17, 59], so we explored if there are also changes in the phases of reprogramming in the transformed lines. We performed RNA-seq during reprogramming in the early phase (D3, D6), the mid-phase (D9, D12), and the late phase (D15). These time points roughly correspond to three waves of gene expression labeled initiation, maturation, and stabilization [17, 59]. The same waves were observed in our data, based on sets of genes specific to the three phases (Fig. 4a, S6a). Interestingly, different transformed lines completed different phases of reprogramming and appeared to fail at distinct stages. Lines that can successfully be reprogrammed (Hdac7SA.Mef2d, Bcl2.Myc, and Hdac7SA.Myc) closely resembled the wildtype MEF reprogramming process (Fig. 4a). Hdacs7SA.E1A and p53DD.E1A progressed through maturation but struggled to upregulate stabilization phase genes (Fig. 4a), although both can ultimately generate small numbers of GFP+ cells (Fig. 2b). The remaining transformed lines failed to reprogram at different stages. SV40T and Hras.E1A failed to upregulate maturation genes, but completed initiation, whilst Hras.Myc failed maturation and stabilization (Fig. 4a, S6).

The mesenchymal-epithelial transition (MET) is a key part of the early stage of reprogramming [59, 60]. All of the cell lines expressed mesenchymal marker genes (Fig. 4b), and all transformed lines managed to navigate the MET, except for Hras.Myc, and to a lesser extent SV40T (Fig. S6b). Interestingly, although Hras.E1A completed the MET, it started with premature upregulation of epithelial genes, for example, *Epcam* (Fig. 4b, S6b). Considering that the three lines that fail to reprogram, Hras.E1A, Hras.Myc and SV40T all have MET progression problems suggests that disrupting the MET impairs the reprogramming of transformed cells.

We next looked at changes in the overall phases of reprogramming in the early, middle, and late stages of reprogramming. We measured the number of significantly deregulated genes at each time point by performing differential expression versus the WT reprogramming on the same day. This can act as a proxy score for the divergence from a typical reprogramming time course. In this analysis, downregulated genes represent genes that fail to upregulate at the correct time point, whilst upregulated genes represent those genes that are erroneously high during reprogramming (Fig. 4c). As expected, the lines that reprogrammed at the highest efficiency (p53DD.Myc) also had the lowest overall divergence, and only diverged from WT reprogramming at day 0 (Fig. 4c). Lines that could reprogram, but at low efficiency (Hdac7SA.Mef2d, Bcl2.Myc, Hdac7SA.E1A, and Hdac7SA.Myc) tended to have high initial divergence from the MEF state (day 0 and 3), but would correctly regulate reprogramming-associated genes at the later time points (day 12 and 15). Finally, lines that fail to reprogram (Hras.E1A and Hras.Myc), or reprogram exceptionally rarely (Bcl2.E1A), tended to have high divergence at all time points (days 0-15). The exception to these patterns was the SV40T line, which followed the reprogramming gene expression program closely, but would diverge at day 12 (Fig. 4c). Interestingly, GO analysis of the downregulated genes (genes that should be upregulated at that specific time point) highlighted the regulation of DNA repair genes, which were defective in both Hras.E1A and SV40T cell lines (Fig. 4d). This data indicates that transformation-specific effects drive line-specific blocks on reprogramming in a stage-dependent manner.

### Chemical intervention can convert some lines from reprogramming-incapable to reprogramming-capable

**Fig. 5:**
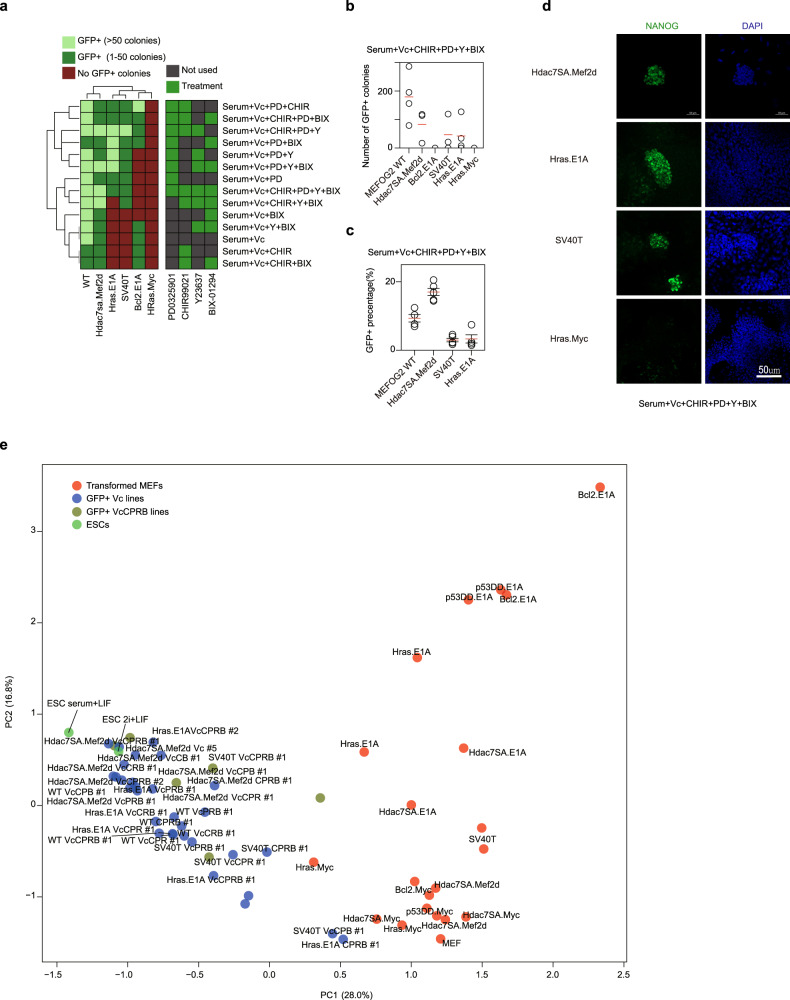
Chemical inhibition can convert some reprogramming-incapable immortalized MEFs to reprogramming-capable. **a** Heatmap showing the number of GFP colonies produced depending upon the reprogramming conditions used, for the indicated transformed lines. Reprogramming experiments were performed between two and five biological replicates for each combination of small molecule inhibitors (See Supplementary Table 1 and Fig. S7b). **b** Chart showing the number of GFP+ colonies in the indicated immortal cell lines with the inhibitor cocktail. Data are from four biological replicates (MEFOGF WT) or three biological replicates (all other transformed cell lines). **c** Percentage of cells sorted by FACS at day 12 of the reprogramming assay in the indicated transformed cell lines or wildtype MEFs. Data are from four biological replicates. **d** Fluorescence microscopy images for NANOG at day 15 of a reprogramming time course for the reprogramming of immortalized ICR-background MEFs cells when treated with the inhibitor cocktail combination Vitamin C (Vc) CHIR99021 (CHIR) PD0325901 (PD) Y23637 (Y) and BIX-01294 (BIX). Scale bar = 50 µm. Images were taken from two biological replicates with three images per slide. **e** PCA of the RNA-seq from the immortalized MEFs, and the reprogrammed iPSCs and ESCs. The various chemical cocktails used during the reprogramming are abbreviated for clarity. Key: Vc = Vitamin C; P = PD0325901; C = CHIR99021; R = Y23637; B = BIX-01294.

The analysis above suggested several pathways that could be manipulated to convert reprogramming-incapable to -capable, specifically, the MET, ribosome biogenesis, cell proliferation, cell adhesion, DNA repair, and apoptosis. To attempt to convert reprograming-incapable to -capable, we screened the effect of a range of inhibitors on three reprogramming-capable lines (WT, Hdac7SA.Mef2d, and Bcl2.E1A MEFs) versus three reprogramming-incapable lines (Hras.E1A, Hras.Myc and SV40T MEFs). We chose to exclude p53DD.Myc from this analysis as although it reprogrammed with high efficiency (Fig. 2b), the p53DD transgene was silenced (Fig. S2e), suggesting that its transformation mechanism is complex. In total, we used 25 inhibitors targeting a range of pathways (Fig. S7a). Whilst most inhibitors had no effect or would ablate reprogramming, 5 inhibitors promoted the reprogramming of one or more of the transformed reprogramming-incapable lines (Fig. S7a). The five small molecules identified targetted: MEK1/2 (PD; PD0325901), GSK3 (CHIR; CHIR99021), ROCK (Y; Y23637), G9a (BIX; BIX-01294), and histone deacetylases (TSA). We tested the inhibitors in combinations and found that the most efficient combination was CHIR, PD, and Y (Fig. 5a, S7b). Interestingly, cocktails including PD and CHIR could also improve wildtype MEF reprogramming, in agreement with previous observations [61]. Most transformed cell lines responded to both PD and CHIR, however, PD-alone was able to convert SV40T and Hras.E1A to reprogramming-capable, as serum+Vc+PD alone resulted in a small number of GFP+ cells (Fig. S7b). Conversely, CHIR, Y, and BIX alone were incapable of converting Hras.E1A MEFs to reprogramming-capable (Fig. S7b). Interestingly the inhibitor cocktails had effects on reprogramming in a line-specific manner, as the addition of PD to the reprogramming cocktail converted the normally reprogramming-capable Bcl2.E1A line to incapable. This highlights the line-specific nature of the reprogramming barriers and shows that overcoming a barrier in one line can potentially initiate a barrier in another transformed line.

We confirmed the resulting iPSC-like cells derived from Hras.E1A or SV40T MEFs reprogrammed with the PD, CHIR, Y, and BIX adopted a normal morphology, expressed pluripotent marker genes by immunofluorescence, qRT-PCR and Western blot (Fig. 5d, and Fig. S7c, d). We sorted the GFP+ cells and allowed the cells to mature through two passages to establish iPSC lines. Gene expression was measured using RNA-seq, and their gene expression was closely correlated with ESCs by both co-correlation and PCA (Fig. 5e and Fig. S8a, b). The results indicate that transformation-specific pathways are impairing the ability to reprogram. Overall, MEK inhibition by PD was the dominant factor in converting reprogramming-incapable to capable.

### Epigenetic defects underly the inability to reprogram

We next focused on three specific cell lines: Hdac7SA.Mef2d, Hras.Myc and Hras.E1A. Hdac7SA.Mef2d could reprogram in serum under normal conditions, whilst Hras.E1A lines were initially resistant to reprogramming (Fig. 2b), but the addition of PD converts it to reprogramming-capable (Fig. 5a–d). Hras.Myc, conversely, could not be converted to reprogramming-capable with any of the conditions we tried (Fig. 5b).

We explored the epigenetic regulation of the transformed MEFs. As a proxy for overall epigenetic activity, we looked at the expression levels of epigenetic factors involved in activation, repression, and the reading of epigenetic marks, as defined by the Epifactors database [62]. As expected, epigenetic-related factors were uniformly significantly upregulated in ESCs compared to MEFs (Fig. S9a), reflecting their more complex epigenetic regulation [63]. For the transformed MEFs, activators were more often significantly upregulated, compared to untransformed MEFs (Fig. S9a). Erasers and readers were only upregulated in several transformed lines, all containing E1A: Hras.E1A and Bcl2.E1A and p53DD.E1A (Fig. S9a). Nonetheless, whilst the different classes of epigenetic regulators varied across the transformed lines and did not discriminate reprogramming-capable from incapable, the general pattern for the majority of lines was increased expression of epigenetic regulators.

**Fig. 6:**
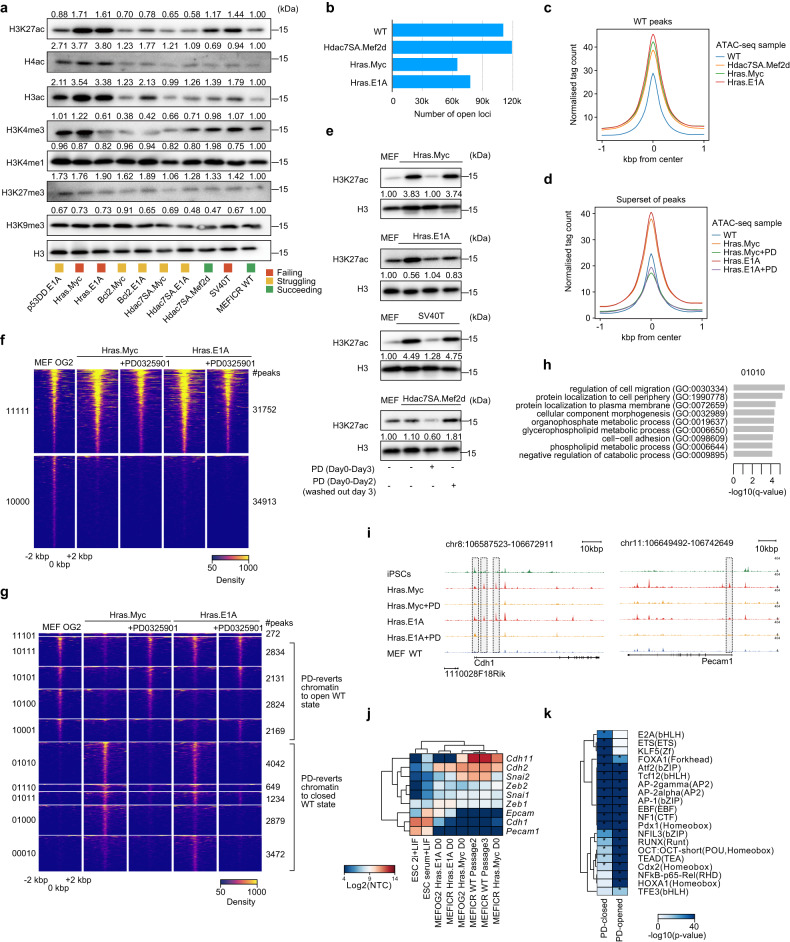
Inhibition of MEK repairs some epigenetic defects in the transformed MEFs. **a** Western blot for a selection of epigenetic marks and total histone H3 as a loading control. The experiment was performed twice with similar results. **b** Bar chart showing the number of ATAC-seq loci identified as open/accessible in the indicated wildtype or transformed MEFs. **c** Pileups of the ATAC-seq data centered on all wildtype MEF peaks and showing the flanking 1 kbp (kilo base pairs). Normalized tag counts (reads per million per bin) are shown for the indicated samples. **d** Pileups of the ATAC-seq data centered on a non-redundant superset of all peaks from all of the ATAC-seq samples. The plot is centered on the peak summit and shows the flanking 1 kbp. Normalized tag counts are shown for the indicated samples. **e** Western blot of activatory histone marks upon addition of PD to the MEFs for 3 days, or cells treated with PD for two days and then washed out on the third data (washed out on day 3). The experiment was performed twice with similar results. **f** Heatmap of the ATAC-seq data showing the permanently open (11111), and open only in the wildtype MEFs (10000). The number of peaks in each class is indicated on the right-hand side. Read density is shown centered on the peak summit and 2 kbp on each side. The full heatmap containing all groups is in Fig. S9b. **g** Heatmap of selected peak groups for the indicated ATAC-seq samples. The full heatmap containing all groups is in Fig. S9b. **h** Gene ontology analysis for nearby genes (a TSS within 10 kbp of an open locus) from the 01010 group (closed in wildtype MEFs, PD causes these loci to close in the transformed MEFs) from Fig. 6g. **i** Genome pileup view of the ATAC-seq data showing the *Cdh1* and *Epcam* loci in wildtype MEFs, Hras.E1A, and Hras.Myc MEFs with or without treatment with PD. Genome assembly is mm10. Regions of open chromatin that are modified in the PD-treated cells are indicated by vertical grey bars. **j** Expression heatmap of selected epithelial and mesenchymal genes in the indicated MEF lines and ESCs. **k** Motif analysis of selected PD-closed and PD-open genes.

Western blot of repressive histone modifications (e.g. H3K27me3, H3K9me3) tended to stay the same in the different lines (Fig. 6a). However, histone acetylation inversely correlated with reprogramming capability as H3K27ac, H3ac, or H4ac was high in reprogramming-incapable lines (Hras.Myc, Hras.E1A and SV40T). This result is somewhat contradictory. Acetylation of histones leads to relaxed chromatin and higher gene expression and is overall beneficial for reprogramming [46, 64, 65]. However, histone acetylation is both context and stage-specific, and increased acetylation at somatic genes can impede reprogramming in the initial stages [43]. Considering the experiment in Fig. 6a is in the transformed MEFs and not during reprogramming, it suggests that a high starting acetylation may be deleterious. These results support epigenetic deregulation as a feature of the transformed lines and suggest increased epigenetic activation through histone acetylation is a feature of transformed reprogramming-incapable MEFs that impairs reprogramming progression.

### Transformed MEFs have a hyperactive chromatin state which MEK-inhibition partially reverts to wildtype

To explore changes in the epigenetic state of the MEFs we performed ATAC-seq on three transformed lines: Hdac7SA.Mef2d (reprogramming-capable), Hras.E1A (initially incapable, but can be induced to reprogram with MEK inhibition), and Hras.Myc (reprograming-incapable) and compared them to wildtype MEFs. Heatmaps of open and closed chromatin indicated, surprisingly, that the Hdac7SA.Mef2d lines were distal from all of the other transformed cell lines and had the greatest number of specific peaks (Fig. S9b, Fig. 6b). The loci that were opened or closed in the Hdac7SA.Mef2d lines were significantly correlated with matching changes in gene expression (Figure S9c). Intriguingly, the overall ATAC signal was substantially higher in all three transformed cell lines compared to wildtype MEFs (Fig. 6c), and this effect was despite fewer open loci in the Hras.Myc and Hras.E1A lines (Fig. 6b). This suggests that the transformed MEFs have increased levels of open chromatin.

We next focused on the Hras.Myc and Hras.E1A lines, and removed the Hdac7SA.Mef2d from further analysis as it is capable of reprogramming under normal conditions. Hras.Myc and Hras.E1A are interesting as they have relatively similar ATAC-seq patterns (Fig. S9b), yet Hras.E1A can be converted to reprogramming-capable, whilst Hras.Myc cannot. The transgenes independently were not barriers themselves to reprogramming, as Hras lines can reprogram (Hras.E1A), Myc lines were reprogramming-capable (Bcl2.Myc and Hdac7SA.Myc), as were E1A transformed lines (Hdac7SA.E1A). Hence, the individual transgenes are not incompatible with reprogramming, only the specific combinations of Hras.E1A and Hras.Myc. One key difference between Hras.E1A and Hras.Myc is that only Hras.E1A upregulated initiation-stage genes and underwent an MET (Fig. 4a, d), suggesting the impairment of reprogramming is already primed in the starting transformed MEFs.

To explore the underlying differences, we performed ATAC-seq on Hras.Myc and Hras.E1A lines with and without PD treatment, and compared them to wildtype MEFs. Interestingly, the global upregulation of the ATAC-seq signal at open loci was lost in the PD-treated samples and returned to levels similar to the wildtype (Fig. 6d). Western blot of H3K27ac, a mark of open active chromatin, supported this observation as H3K27ac was higher in the transformed MEFs and treatment with PD for 3 days reduced H3K27ac to wildtype levels (Fig. 6e). Interestingly the result was transient, as a treatment for 2 days followed by washing out reverted H3K27ac levels to the hyperactive state (Fig. 6e). This suggests that when PD inhibits MEK it leads to the indirect closing of chromatin and reductions in the global levels of H3K27ac. However, this effect was transient, and incomplete as the overall epigenetic landscape did not revert to a wildtype state, and 34,913 loci open in wildtype MEFs remained closed in the transformed cell lines (Fig. 6f).

To explore the mechanisms underlying the action of PD, we next divided the loci into two types based upon their state in wildtype MEFs and the effect of PD: (1) those loci that PD opens and reverts to a wildtype state, and (2) those loci that PD closes, reverting to the wildtype state (Fig. 6g). There was a roughly equal split between the two classes (10,230 peaks versus 12,276 peaks; Fig. 6g). GO analysis of the peaks that were specifically reverted to wildtype state in the Hras.Myc cells suggested that cell-cell adhesion gene loci were being remodeled (Fig. 6h). Indeed, chromatin at *Cdh1* and *Epcam* (two epithelial genes) were open in the Hras.E1A and Hras.Myc MEFs, but were closed when the cells were treated with PD and reverted to a wildtype MEF pattern at these genes (Fig. 6i). Importantly, *Cdh1*, *Epcam*, and other epithelial genes were not expressed in the transformed MEFs (Fig. 6j), indicating that epigenetic regulation at epithelial gene loci is deleterious for reprogramming of transformed cell lines.

We next looked at possible downstream targets of MEK signaling that mediated this indirect effect on chromatin. We used motif analysis to look for changes in transcription factor occupancy at PD-modulated peaks (Fig. 6k). Interestingly, peaks that were closed in response to PD treatment were specifically enriched with ETS, E2A, and KLF motifs. KLF proteins are major regulators of MET processes and are pluripotent factors, whilst ETS factors have been identified as barriers to wildtype untransformed reprogramming [66]. Loci that PD treatment opens were enriched with REL, HOX, and TFE family TFs (Fig. 6k). To explore deeper we looked at two small groups of chromatin loci that distinguished reprogramming-capable from -incapable, 10001 (specifically open in WT and Hras.E1A + PD) and 01110 (specifically closed in WT and Hras.E1A + PD) (Fig. 6g). Interestingly, GO analysis of the 01110-group suggested that enhancers near genes related to cell differentiation were erroneously opened (Figure S10a), for example at the developmental-related genes *Isl1* and *Tcf7l2* (Figure S10b). This was an effect on the chromatin state as *Isl1* and *Tcf7l2* were undetected in the RNA-seq data. This pattern extended to all of the genes in the vicinity of the 01110-chromatin state which were not significantly up or downregulated (Fig. S10c). Conversely, the genes associated with the 10001 group were significantly downregulated (Fig. S10c), although GO analysis suggests they are related to basic cellular processes such as cytoskeleton organization (Fig. S10d). Overall, this suggests the epigenetic activation of developmental genes is deleterious for reprogramming, and MEK inhibition can indirectly modulate the epigenetic state to close chromatin at developmental-related genes.

## Discussion

Reprogramming cancerous/transformed cells to pluripotent cells, followed by their differentiation, could form a valuable experimental model to recapitulate the earliest stages of tumorigenesis [67, 68]. However, reprogramming primary cancerous tissues is often difficult or impossible for many cancers, and even when possible the efficiency is considerably less than wildtype cells [31]. Additionally, there remain doubts about the bona fide reprogramming of cancerous cells [32, 38, 39]. A significant problem is the heterogeneity of primary cancer cells. To overcome this problem, we focused on isogenic transformed MEF lines that allow us to control cell type heterogeneity.

There is an intimate but unclear link between reprogramming and transformation [1, 68]. This link was recently explored in a novel system with multiple inducible genes that could be manipulated to either transform wildtype MEFs or perform reprogramming to iPSCs [20]. This elegant study showed that reprogramming and transformation followed similar initial paths, but then later diverged. The system they used for transformation was K-ras^G12D^ and *Myc* overexpression [20], which is similar to the Hras.Myc (*Hras*^*G12V*^/*Myc*) line described in this manuscript. Intriguingly, Hras.Myc is not compatible with reprogramming, and fails very early in the reprogramming process. However, several other lines described here can be transformed and remain compatible with reprogramming. Potentially, transformation strategies that remain compatible with reprogramming may recapitulate more of the reprogramming process as they transform and only diverge from reprogramming at later stages.

*Ras* overexpression is beneficial for reprogramming wildtype MEFs, but is a major barrier to reprogramming transformed cells [69]. Indeed, when Hras^G12V^ was overexpressed in p53-null immortalized MEFs reprogramming was blocked [69]. This is reminiscent of the effect in the Hras.Myc and Hras.E1A lines, which were also resistant to reprogramming. However, we show that inhibition of MEK by PD enables Hras.E1A cells to reprogram, whilst Hras.Myc cells remain reprogramming-incapable. This indicates that the reprogramming of Hras^G12V^ is context-dependent.

Several studies have identified small molecules that assist in reprogramming transformed cells to a pluripotency. mTOR was identified as a barrier to the reprogramming of sarcoma cells [70]. In our experiments, mTOR inhibition had no effect. This again highlights the context-specific nature of the barriers in different transformed cell lines. Overall, MEK inhibition was the only essential molecule, which agrees with a previous study that showed that MEK inhibition assisted in reprogramming transformed human cell lines [71]. PD is also a key component of the 2iLIF medium used to maintain mouse ESCs in a naïve ground state [61, 72]. It is unclear if PD plays the same role in assisting in reprogramming transformed MEFs and in promoting pluripotency. Indeed, reprogramming with PD is deleterious for reprogramming wildtype MEFs, at least in the early stages [69, 73]. The SV40T transformed line was an interesting case in this regard, as it could be converted to reprogramming-capable, and only required the addition of PD, although reprogramming efficiency could be improved with the addition of other small molecule inhibitors. We speculate that SV40T is similar to Hras.E1A, and both cell lines can correctly initiate reprogramming but fail to mature or stabilize, both late-stage processes. The addition of PD appears to be sufficient for reprogramming as long as the initial stages can be overcome.

Ultimately, our model suggests the context-specific reprogramming of transformed cell lines. Different transformations lead to diverse blocks at different stages. Inhibition of ERK by PD can overcome some of these barriers, primarily in Ras-transformed cells [69, 71]. However, each transformed cell line can harbor barriers that block reprogramming at unique stages. This perhaps reflects the diversity of regulatory features disrupted in cancer and may relate to the ‘stemness’ of a cancer type or method of transformation [74]. Nonetheless, insights into reprogramming can inform the process of transformation and provide new models to address tumorigenesis.

## Methods summary

### Animal experimental approval

Ethical approval for the generation of MEFs and teratoma assays was granted by the Southern University of Science and Technology animal ethical committee, approval number: SUSTC-2019-005.

### Cell culture and MEF transformation

MEFs were derived from E13.5 embryos from OG2 mice [43] (MEFOG2) or were from a wildtype ICR genetic background (MEFICR). The sex of the animals was unknown. MEFOG2 cells contain an Oct4-GFP reporter, as described in [43]. The MEFICR cells did not contain a reporter. All of the experiments were performed using the MEFOG2 lines, except for the experiments in Fig. 5d, S2b, d–f, h-j, all of S3, and S5d which used the MEF ICR lines. MEF cells were cultured in DMEM high-glucose media containing 10% FBS (GIBCO), 1 × GlutaMAX (GIBCO), and 1x nonessential amino acids (GIBCO), 0.5x Penicillin/Streptomycin (Hyclone). We generated immortalized MEF cell lines by transfecting the following transgenes singly or in combination: P53DD, PMM2-SV40T, PMM2-Hdac7SA, PMM2-Mef2d, Bcl2, PMM2-Myc, Ras, PMM2-E1A. Immortalized MEFs were passaged for at least a month to remove wildtype untransformed MEFs. 3T3-L1, 4T1 and N2a cells were from the ATCC collection.

### Somatic cell reprogramming

Wildtype MEFs and transformed MEFs in an OG2 reporter background were reprogrammed as described in [43]. Briefly, a polycistronic OKSM [75], lentivirus was transfected into 15,000 MEFs in one well of a 12-well gelatin plate. One day after transfection the medium was changed to reprogramming medium and cultured for 15 days: DMEM high-glucose media containing 15% FBS (GIBCO), 1× GlutaMAX (GIBCO), 1x non-essential amino acids (GIBCO), 0.5x Penicillin/Streptomycin (Hyclone), 1 mM sodium pyruvate (GIBCO), 0.1 mM 2-mercaptoethanol (GIBCO), 1000 U/ml leukemia inhibitory factor (LIF) (Millipore). Vitamin C (Vc) (Sigma) was used in the majority of reprogramming media, unless indicated, and was added at a concentration of 50 μg/ml. The medium was changed daily. Inhibitors were added when the medium was changed to the reprogramming medium: PD0325901 (1 μM, Sigma), CHIR99021 (3 μM, Sigma), Y23637 (10 μM, Sigma), BIX-01294 (200 nM, Selleck), and TSA (50 nM, Sigma). iPSC lines were established by allowing the GFP+ cells to pass through a further two passages.

### Karyotype analysis

When the immortalized MEF ICR line cell growth density reached 80 to 90%, colchicine (0.2 μg/ml, Selleck), was added to the culture medium to a final concentration of 0.2 μg/ml. The cells were incubated at 37 °C for 120 min. After colchicine treatment, cells were washed twice with PBS, and 0.5 ml of 0.25% trypsin was added for digestion. Then, 7 ml of 0.075 mol/L KCl solution preheated to 37 °C was added, and the cell suspension was blown with a straw and incubated at 37 °C in a water bath for 25 min. Collected cells were fixed with a fixing solution (methanol glacial acetic acid 3:1 preparation) at 37 °C, 3 min. After fixation, cells were centrifuged at 1200 revolutions per min (rpm) for 5 min, after which the supernatant was discarded. 7 ml of fresh fixing solution was added, and the cells were gently beaten with a pipette tip, and fixed in a 37 °C water bath for 40 min. Cells were centrifuged at 1200 rpm for 5 min, after which the supernatant was discarded. Subsequently, 7 ml of fresh fixing solution was added, followed by gentle beating with a filter tip. The cells were fixed in a 37 °C water bath for 40 min. After fixation, the cells were centrifuged at 1200 rpm for 5 min, and then most of the fixation solution was removed, and the cells were resuspended with part of the fixation solution. Suspended cells were pipetted onto a slide from a distance of about 30 cm. Immediately after dropping the cells on the slide, the slide was incubated at 75 °C and baked for 3 h. Next, 0.03 g trypsin powder to 55 ml normal saline was added, followed by a gentle shaking, before adjusting the pH to about 7.2 with 3% Tris-HCl. The preparation was put into trypsin digestion solution for 8–10 s, followed by the addition of saline to stop digestion, Giemsa staining (C0133, Beyotime Biotech) solution was added for 5–10 min. Both sides were gently rinsed with running water and allowed to dry at room temperature. After the slides dried, they were examined under a microscope to look for a good chromosome split.

### Teratoma formation

5–10 million mouse iPSCs in a slurry of Matrigel and mTeSR (1:1) medium were injected into 8-week-old female nude (BALB/cNj-Foxn1^nu^/Gpt) immunodeficient mice. Teratoma growth was quantified by measuring the approximate elliptical area (mm^2^) with calipers measuring the outward width and height after growth for 60 days. Representative tumors were dissected and sectioned and slices were stained with hematoxylin and eosin.

### Alkaline phosphatase staining

Reprogrammed cells were stained with alkaline phosphatase according to the manufacturer’s protocols. In brief, reprogramming cells were fixed in 1% (w/v) formaldehyde, and then cells were stained with BCIP/NBT Alkaline Phosphatase Color Development Kit (C3206, Beyotime Biotech) according to the kit’s instructions.

### Flow cytometry

Reprogramming cells were digested with trypsin and washed with DPBS once, and analyzed or sorted with a BD FACS Aria III flow cytometer. To monitor cellular reprogramming status, reprogrammed cells were stained with antibodies: anti-CD44-PE (25-0441-82, 1:500, Thermo Fisher) and anti-ICAM-1-FITC (sc-8439 FITC, 1:200, Santa Cruz Biotechnology). Cells were sorted and analyzed on a BD FACS Aria III instrument, with the following gain settings: For cancer cell line (Fig. 1c): FSC 128; SSC 254; FITC 495; PE-cy7 478. For immortalized MEFs GFP detection (Fig. 5c) FSC 166; SSC 263; FITC 310.

### Western blot

Cells were lysed with RIPA buffer (P0013B, Beyotime Biotech). Proteins were resolved in sodium dodecyl sulfate-polyacrylamide gel electrophoresis (SDS-PAGE; 15% (w/v) for blots involving histones and 12% (w/v) for all other proteins) and transferred onto pre-activated polyvinylidene fluoride (PVDF) membranes (IPVH00010, Millipore, MA, USA). The PVDF Membranes were incubated with anti-H3 (ab1791, 1:3000, Abcam), anti-H3K27ac (ab4279, 1:2000, Abcam), anti-H3ac (06-599,1:2000, Millipore), anti-H4ac (ab46983, 1:2000, Abcam), anti-H3K4me3 (ab8580, 1:2000, Abcam), anti-H3K4me (ab176877,1:2000, Abcam), anti-H3K27me3 (07-449,1:2000, Millipore), anti-H3K9me3 (ab8898, 1:2000, Abcam), anti-NANOG (8822 S, 1:1000, cell signaling), anti-OCT4 (sc-5279, 1:1000, Santa Cruz Biotechnology), anti-P53 (sc-126, 1:1000, Santa Cruz Biotechnology), anti-HDAC7 (ab12174,1:2000, Abcam), anti-MEF2D (ab32845,1:2000, Abcam), anti-ACTIN (ab8227, 1:3000, Abcam), anti-MYC (ab32074,1:2000, Abcam), anti-SOX2 (4900S, 1:1000, cell signaling), anti-MDM2 (AF7499,1:1000, Beyotime), anti-CNK1A (AF5252,1:1000, Beyotime) anti-Phospho-p53(Ser15) (AF5893,1:1000, Beyotime). Afterward, the membranes were incubated with HRP-conjugated goat antirabbit IgG (ab205718, 1:3000, Abcam) and visualized using an enhanced chemiluminescence BeyoECL method (P0018AS, Beyotime Biotech) on a Tanon 6100C machine. All Uncropped unprocessed western blots are in Supplementary Material.

### Immunofluorescence

Cells were washed with cold PBS and fixed using 4% (w/v) paraformaldehyde in PBS for 10 min at room temperature. Then cells were washed with PBS and permeabilized with 0.25% (v/v) Triton X-100 for 10 minutes at room temperature. Fixed cells were then washed with PBS three times and blocked with 3% (w/v) bovine serum albumin for 1 hour at room temperature. Then cells were incubated with primary antibodies: anti-NANOG (8822 S, 1:1000, cell signaling), at 4 °C overnight in primary antibody. Following overnight incubation, cells were washed with wash buffer (0.1% (v/v) Tween-20 in PBS) and incubated with secondary antibodies Alexa Fluor series (Life Technologies) in wash buffer for 1 h at room temperature. Cells were washed again and nuclei were stained using DAPI (5 μg/ml, Thermo). Then we used a fluorescent microscope (LSM980 Carl Zeiss) to capture at least three slides for each sample at ×100 magnification. Laser lines 488, 568, and 405 nm were used to stimulate and observe Nanog, tdTomato, and DAPI. Immunofluorescence imaging experiments were performed in biological duplicate, and at least three views were gathered per slide

### RT-qPCR

Total RNA from cells was isolated using RNAzol RT (MRC, RN190) according to the manufacturer’s protocols. cDNA synthesis by using a PrimeScript RT Master Mix (Takara, RR036A). Real-time PCR was performed in triplicate using SYBR Premix Ex Taq (Takara, RR820A) and using a Biorad Real-time PCR system. The primers used are listed in Supplementary Table S3.

### RNA-seq preparation and analysis

RNA was isolated using RNAzol RT (MRC, RN190) according to the manufacturer’s protocol and prepared for sequencing with RNA-seq NEB Next Ultra RNA Library Prep Kit (NEB, #7530). RNA quality was tested using an Agilent 2100 and a minimum RIN score of 8.0 was required for sequencing. Samples were sequenced on an Illumina Novaseq 6000.

RNA-seq was analyzed essentially as described in [52], except scTE/te_counts was used to assign mapped reads to genes and TEs [76], and the UCSC genome browser repeat mask track and GENCODE vM23 annotation was used for TE and gene assignment. Differential expression was determined using DESeq2 [77]. A gene was considered differentially expressed had an absolute fold-change of at least 2, and a Bonferroni-Hochberg corrected p-value of 0.01. GSEA was performed using fgsea [78], and GO was done with goseq [79]. Cell type determination was scored with DPre [53]. Other analyses were performed using glbase3 [80].

### ATAC-seq preparation and analysis

ATAC-seq library was generated using the Tn5 enzyme from the TruePrep DNA Library Prep Kit V2 (Vazyme, TD501-02) as previously described [81]. Briefly, a total of ~50,000 cells were washed once with 50 μl of cold PBS and resuspended in 50 μl lysis buffer (10 mM Tris-HCl pH 7.4, 10 mM NaCl, 3 mM MgCl2, 0.2% (v/v) IGEPAL CA-630). The nuclei were centrifuged for 10 min at 500 × *g* at 4 °C, followed by the addition of 50 μl transposition reaction mix (25 μl TD buffer, 2.5 μl Tn5 transposase (Vazyme), and 22.5 μl nuclease-free water). Samples were PCR-amplified and purified using a MinElute kit (Qiagen). After selecting an appropriate PCR cycle number (See [81]) samples were sequenced on an Illumina sequencer. ATAC-seq data was analyzed essentially as described in reference: [66]. Briefly, reads were aligned to the mm10 mouse genome with bowtie2 [82], peaks were called with MACS2 [83], and then the *redefine_peaks* function, which is a generalized reimplementation of the algorithm in [66], was used to recover low-scoring peaks by sharing peak information across samples [84]. DNA binding motifs were detected using HOMER [85]. All other analyses were performed using glbase3 [80]. ATAC-seq data from GSE93029 [66] and GSE103980 [86] were reanalyzed as part of this study.

### Statistical analysis, reporting, and biological replication

No statistical test was used to determine the sample size. Animal experiments were performed in biological duplicates, and there was no randomization performed to select animals for experimentation. The investigator was not blinded to the experimental details.

Differential gene expression was calculated using DESeq2 (v1.36.0). A gene was considered significantly differentially regulated if it had an absolute fold-change of at least 2 and a Bonferroni-Hochberg corrected *p*-value (*q*-value) of <0.01. This criterion was used in Figs. 3a, 4c, Figs. S1c, and d. RNA-seq experiments were performed in at least biological duplicate (different samples on different days, or independent cell lines). Gene ontology analysis was performed using goseq (v1.48.0) and statistics were calculated using goseq’s internal statistical model. A gene ontology category was considered significantly enriched if there were at least 50 genes in that GO term and a Bonferroni-Hochberg corrected *p*-value (*q*-value) of <0.01. GSEA was performed using fgsea (v1.22.0). Gene sets were considered enriched or depleted if they had an absolute NES (normalized enrichment score) of at least 1.5 and a Bonferroni-Hochberg corrected *p*-value (*q*-value) of <0.01. Transcription factor motif analysis was performed using HOMER. A motif was considered significantly enriched if the uncorrected *p*-value was <0.00001.

Western blots were repeated at least twice with similar results, except for Fig. S7c which was performed once. Figure 6a was repeated three times. FACs analysis (Figs. 1c and 5c) were performed three times with similar results. All qRT-PCR experiments were performed using at least three biological replicates with three technical replicates each. Immunofluorescence imaging was performed in biological duplicate, and at least three views were gathered per slide. In total we generated twenty distinct transformed MEF lines, using ten transgene combinations, in two genetic backgrounds, MEFOG2 and MEFICR. We reprogrammed the MEF OG2 transformed cells to generate 37 iPSC-like lines.

## Supplementary information

Supplementary Material

Supplementary Table 1

Supplementary Table 2

Supplementary Table 3

## Data Availability

The RNA-seq and ATAC-seq data generated as part of this study are available in the gene expression omnibus (GEO) public database under accession number GSE213225.
