# Supplementary material for: Restricting epigenetic activity promotes the reprogramming of transformed cells to pluripotency in a line-specific manner
Source: Cell Death Discov. 2023 Jul 14;9:245. doi: 10.1038/s41420-023-01533-8 (PMC10349098; doi:10.1038/s41420-023-01533-8)
Supplement: Supplementary file 1 — Supplementary Material [file 41420_2023_1533_MOESM1_ESM.pdf]

Supplementary Tables

Supplementary Table 1 – Table of experimental samples and data for each figure in the manuscript, includes raw uncropped western blots.

Supplementary Table 2 – Genes assigned to the 'MEF senescent signature' and the 'reprogramming permissive signature'.

Supplementary Table 3 – Sequences of the transgenes used for aligning the RNA-seq data.

Supplementary Results

Measurement of the transgenes in the RNA-seq data

Transgene expression was measured by aligning the RNA-seq to an index composed of the FASTA sequences for the transgenes used in this study (**Supplementary Table 3**). The HsMYC (human c-MYC; ENST00000259523) measures the expression of the polycistronic OSKM transcript (Warlich et al., 2011). Note that the HsOCT4, HsSOX2 and HsKLF4 transgenes are not detectable in this RNA-seq data as the RNA is cleaved and only the HsMYC has a poly-A tail. GFP/eGFP measures the expression of the GFP reporter from the OG2 reporter (Zhuang et al., 2018).

References

Hutchins, A.P., Yang, Z., Li, Y., He, F., Fu, X., Wang, X., Li, D., Liu, K., He, J., Wang, Y.*, et al.* (2017). Models of global gene expression define major domains of cell type and tissue identity. Nucleic Acids Res *45*, 2354-2367.

Medvedeva, Y.A., Lennartsson, A., Ehsani, R., Kulakovskiy, I.V., Vorontsov, I.E., Panahandeh, P., Khimulya, G., Kasukawa, T., Consortium, F., and Drablos, F. (2015). EpiFactors: a comprehensive database of human epigenetic factors and complexes. Database (Oxford) *2015*, bav067.

Samavarchi-Tehrani, P., Golipour, A., David, L., Sung, H.K., Beyer, T.A., Datti, A., Woltjen, K., Nagy, A., and Wrana, J.L. (2010). Functional genomics reveals a BMP-driven mesenchymal-to-epithelial transition in the initiation of somatic cell reprogramming. Cell Stem Cell *7*, 64-77.

Steffens, S., Fu, X., He, F., Li, Y., Babarinde, I.A., and Hutchins, A.P. (2020). DPre: computational identification of differentiation bias and genes underlying cell type conversions. Bioinformatics *36*, 1637-1639.

Warlich, E., Kuehle, J., Cantz, T., Brugman, M.H., Maetzig, T., Galla, M., Filipczyk, A.A., Halle, S., Klump, H., Scholer, H.R.*, et al.* (2011). Lentiviral vector design and imaging approaches to visualize the early stages of cellular reprogramming. Mol Ther *19*, 782-789.

Zhuang, Q., Li, W., Benda, C., Huang, Z., Ahmed, T., Liu, P., Guo, X., Ibanez, D.P., Luo, Z., Zhang, M.*, et al.* (2018). NCoR/SMRT co-repressors cooperate with c-MYC to create an epigenetic barrier to somatic cell reprogramming. Nat Cell Biol *20*, 400-412.

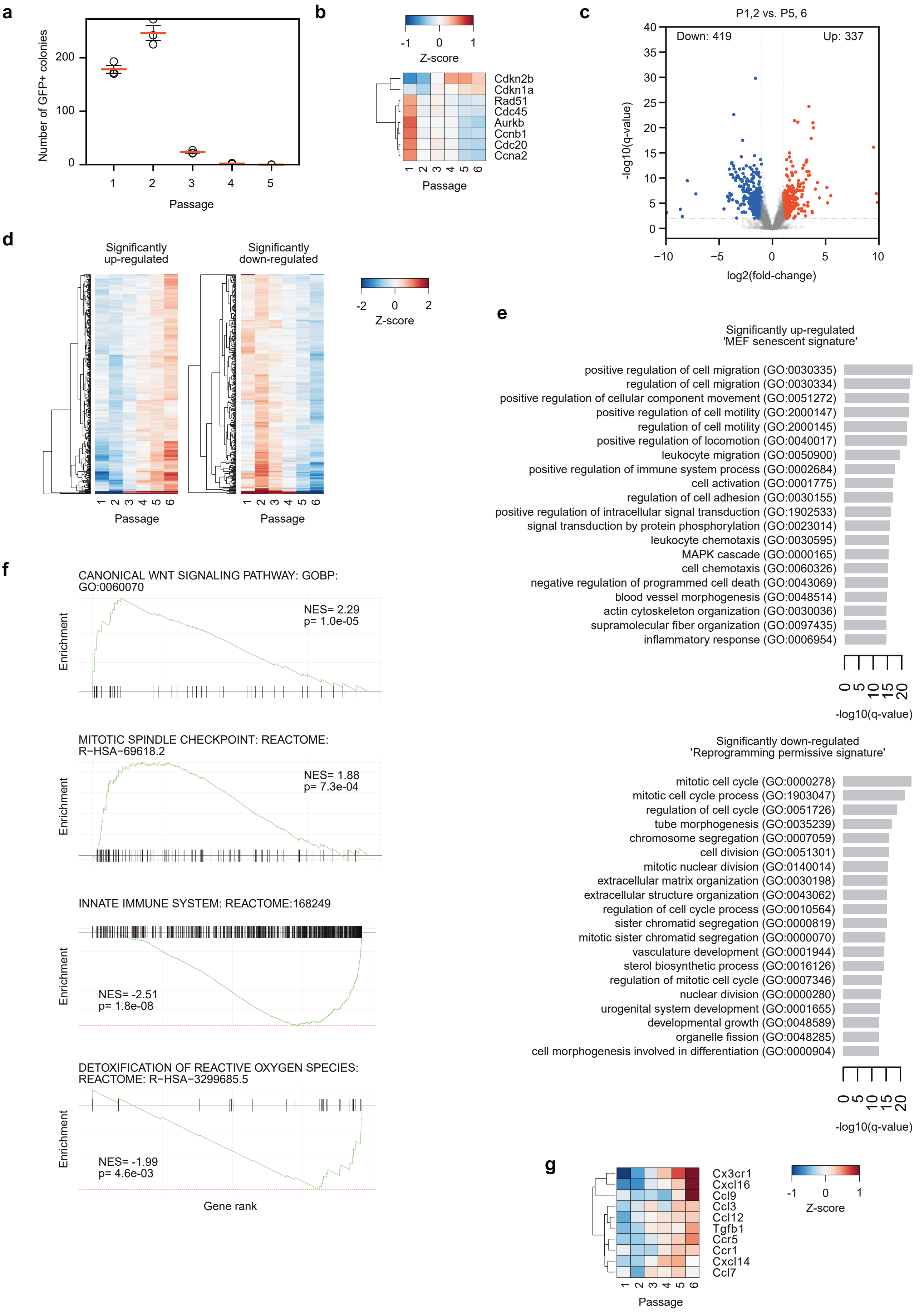

a
Number of GFP+ colonies
Passage
b
Z-score
Cdkn2b
Cdkn1a
Rad51
Cdc45
Aurkb
Ccnb1
Cdc20
Ccna2
Passage
c
P1,2 vs. P5, 6
Down: 419
Up: 337
-log10(q-value)
log2(fold-change)
d
Significantly up-regulated
Significantly down-regulated
Z-score
Passage
Passage
e
Significantly up-regulated 'MEF senescent signature'
positive regulation of cell migration (GO:0030335)
regulation of cell migration (GO:0030334)
positive regulation of cellular component movement (GO:0051272)
positive regulation of cell motility (GO:2000147)
regulation of cell motility (GO:2000145)
positive regulation of locomotion (GO:0040017)
leukocyte migration (GO:0050900)
positive regulation of immune system process (GO:0002684)
cell activation (GO:0001775)
regulation of cell adhesion (GO:0030155)
positive regulation of intracellular signal transduction (GO:1902533)
signal transduction by protein phosphorylation (GO:0023014)
leukocyte chemotaxis (GO:0030595)
MAPK cascade (GO:0000165)
cell chemotaxis (GO:0060326)
negative regulation of programmed cell death (GO:0043069)
blood vessel morphogenesis (GO:0048514)
actin cytoskeleton organization (GO:0030036)
supramolecular fiber organization (GO:0097435)
inflammatory response (GO:0006954)
-log10(q-value)
Significantly down-regulated 'Reprogramming permissive signature'
mitotic cell cycle (GO:0000278)
mitotic cell cycle process (GO:1903047)
regulation of cell cycle (GO:0051726)
tube morphogenesis (GO:0035239)
chromosome segregation (GO:0007059)
cell division (GO:0051301)
mitotic nuclear division (GO:0140014)
extracellular matrix organization (GO:0030198)
extracellular structure organization (GO:0043062)
regulation of cell cycle process (GO:0010564)
sister chromatid segregation (GO:0000819)
mitotic sister chromatid segregation (GO:0000070)
vasculature development (GO:0001944)
sterol biosynthetic process (GO:0016126)
regulation of mitotic cell cycle (GO:0007346)
nuclear division (GO:0000280)
urogenital system development (GO:0001655)
developmental growth (GO:0048589)
organelle fission (GO:0048285)
cell morphogenesis involved in differentiation (GO:0000904)
-log10(q-value)
f
CANONICAL WNT SIGNALING PATHWAY: GOBP: GO:0060070
Enrichment
NES= 2.29
p= 1.0e-05
MITOTIC SPINDLE CHECKPOINT: REACTOME: R-HSA-69618.2
Enrichment
NES= 1.88
p= 7.3e-04
INNATE IMMUNE SYSTEM: REACTOME:168249
Enrichment
NES= -2.51
p= 1.8e-08
DETOXIFICATION OF REACTIVE OXYGEN SPECIES: REACTOME: R-HSA-3299685.5
Enrichment
NES= -1.99
p= 4.6e-03
Gene rank
g
Cx3cr1
Cxcl16
Ccl9
Ccl3
Ccl12
Tgfb1
Ccr5
Ccr1
Cxcl14
Ccl7
Passage
Z-score

Supplementary Fig. 1. Late passage MEFs lose reprogramming capability and become senescent.

a Number of GFP+ colonies from a reprogramming experiment using OCT4KI-MEFs from the indicated passage number. Data is from three biological replicates.

b Heatmap for the expression of selected cell cycle genes in the different passages of MEFs.

c Volcano plot for differentially expressed genes when comparing passage 1, 2 versus passage 5, 6. A gene was considered significantly different if it had an absolute fold-change of 2 and a q-value of less than 0.01 (Bonferroni-Hochberg corrected p-value).

d Heatmaps of the expression of the significantly up or down-regulated genes in the indicated MEF passages.

e Gene ontology analysis of the significantly up and down-regulated genes. A GO term was considered significant at a Bonferroni-Hochberg corrected p-value of less than 0.01. Based on these GO, we labelled the up-regulated genes as the 'MEF senescent signature' and the down-regulated genes as the 'reprogramming permissive signature'

f GSEA for the up and down-regulated genes. Selected Reactome or GO BP (biological process) terms are shown. NES (normalized enrichment score), and the Bonferroni-Hochberg adjusted p-value are indicated.

g Heatmap of selected significantly differentially expressed cytokines and chemokines.

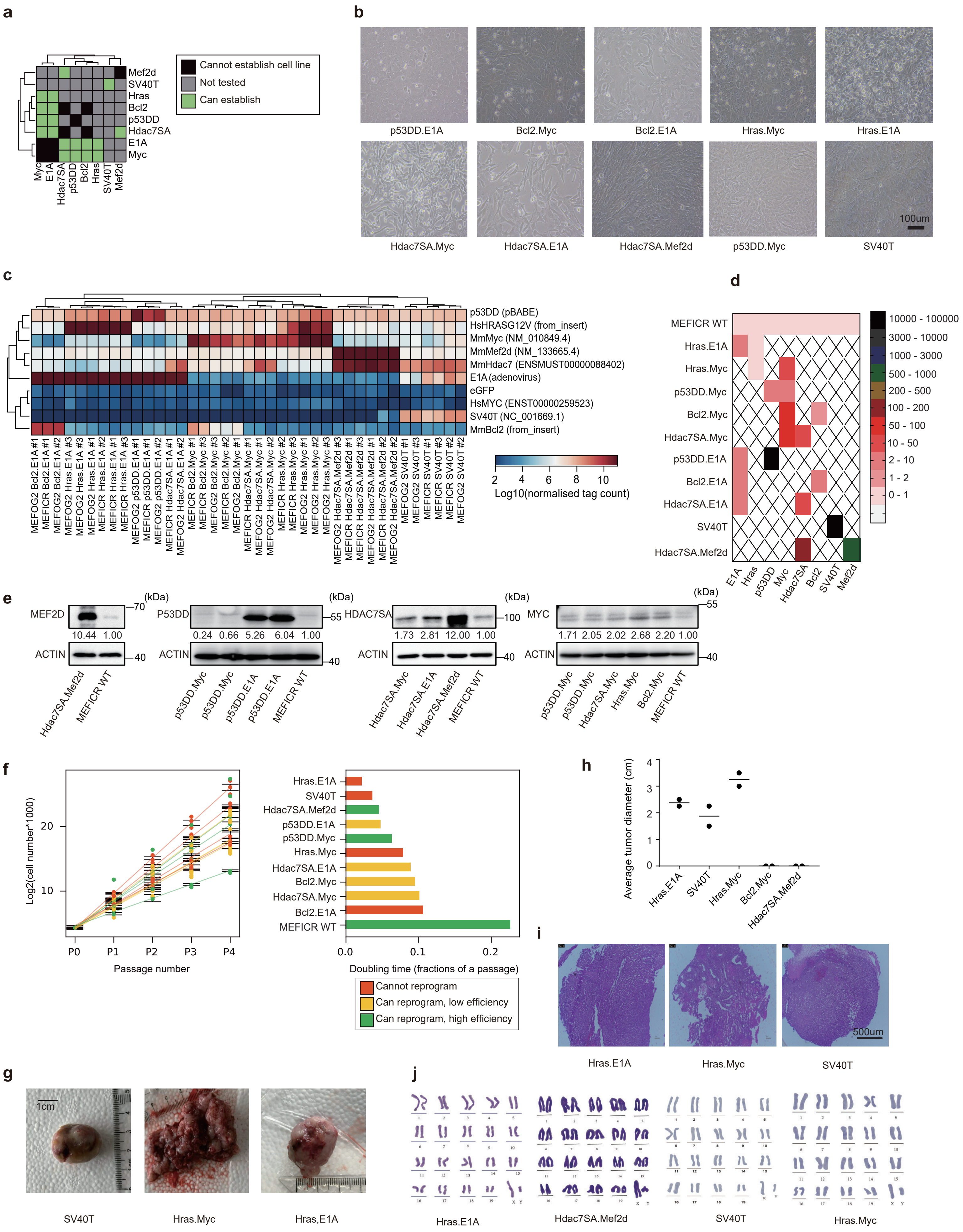
a
Mef2d
SV40T
Hras
Bcl2
p53DD
Hdac7SA
E1A
Myc
Myc
E1A
Hdac7SA
p53DD
Bcl2
Hras
SV40T
Mef2d
Cannot establish cell line
Not tested
Can establish
b
p53DD.E1A
Bcl2.Myc
Bcl2.E1A
Hras.Myc
Hras.E1A
Hdac7SA.Myc
Hdac7SA.E1A
Hdac7SA.Mef2d
p53DD.Myc
SV40T
100um
c
p53DD (pBABE)
HsHRASG12V (from_insert)
MmMyc (NM_010849.4)
MmMef2d (NM_133665.4)
MmHdac7 (ENSMUST00000088402)
E1A (adenovirus)
eGFP
HsMYC (ENST00000259523)
SV40T (NC_001669.1)
MmBcl2 (from_insert)
MEFOG2 Bcl2.E1A #1
MEFICR Bcl2.E1A #1
MEFOG2 Bcl2.E1A #2
MEFOG2 Hras.E1A #3
MEFOG2 Hras.E1A #3
MEFICR Hras.E1A #1
MEFOG2 Hras.E1A #2
MEFICR Hras.E1A #1
MEFICR Hras.E1A #3
MEFOG2 p53DD.E1A #1
MEFICR p53DD.E1A #1
MEFOG2 p53DD.E1A #2
MEFICR Hdac7SA.E1A #1
MEFOG2 Hdac7SA.E1A #2
MEFICR Bcl2.Myc #1
MEFICR Bcl2.Myc #3
MEFOG2 Bcl2.Myc #3
MEFICR Bcl2.Myc #2
MEFOG2 Bcl2.Myc #1
MEFICR Hdac7SA.Myc #1
MEFOG2 Hdac7SA.Myc #1
MEFOG2 Hdac7SA.Myc #2
MEFICR Hras.Myc #2
MEFOG2 Hras.Myc #3
MEFICR Hras.Myc #1
MEFOG2 Hras.Myc #2
MEFOG2 Hras.Myc #3
MEFOG2 Hdac7SA.Mef2d #3
MEFICR Hdac7SA.Mef2d #1
MEFICR Hdac7SA.Mef2d #3
MEFOG2 Hdac7SA.Mef2d #1
MEFICR Hdac7SA.Mef2d #2
MEFOG2 Hdac7SA.Mef2d #2
MEFOG2 SV40T #1
MEFOG2 SV40T #3
MEFICR SV40T #1
MEFICR SV40T #3
MEFICR SV40T #2
MEFOG2 SV40T #2
2 4 6 8 10
Log10(normalised tag count)
d
MEFICR WT
Hras.E1A
Hras.Myc
p53DD.Myc
Bcl2.Myc
Hdac7SA.Myc
p53DD.E1A
Bcl2.E1A
Hdac7SA.E1A
SV40T
Hdac7SA.Mef2d
E1A
Hras
p53DD
Myc
Hdac7SA
Bcl2
SV40T
Mef2d
10000 - 100000
3000 - 10000
1000 - 3000
500 - 1000
200 - 500
100 - 200
50 - 100
10 - 50
2 - 10
1 - 2
0 - 1
e
MEF2D
(kDa)
70
10.44 1.00
ACTIN
40
Hdac7SA.Mef2d
MEFICR WT
P53DD
(kDa)
55
0.24 0.66 5.26 6.04 1.00
ACTIN
40
p53DD.Myc
p53DD.Myc
p53DD.E1A
p53DD.E1A
MEFICR WT
HDAC7SA
(kDa)
100
1.73 2.81 12.00 1.00
ACTIN
40
Hdac7SA.Myc
Hdac7SA.E1A
Hdac7SA.Mef2d
MEFICR WT
MYC
(kDa)
55
1.71 2.05 2.02 2.68 2.20 1.00
ACTIN
40
p53DD.Myc
p53DD.Myc
Hdac7SA.Myc
Hras.Myc
Bcl2.Myc
MEFICR WT
f
Log2(cell number*1000)
20
10
P0 P1 P2 P3 P4
Passage number
Hras.E1A
SV40T
Hdac7SA.Mef2d
p53DD.E1A
p53DD.Myc
Hras.Myc
Hdac7SA.E1A
Bcl2.Myc
Hdac7SA.Myc
Bcl2.E1A
MEFICR WT
0.0 0.1 0.2
Doubling time (fractions of a passage)
Cannot reprogram
Can reprogram, low efficiency
Can reprogram, high efficiency
h
Average tumor diameter (cm)
4
3
2
1
0
Hras.E1A
SV40T
Hras.Myc
Bcl2.Myc
Hdac7SA.Mef2d
i
500um
Hras.E1A
Hras.Myc
SV40T
g
1cm
SV40T
Hras.Myc
Hras,E1A
j
Hras.E1A
Hdac7SA.Mef2d
SV40T
Hras.Myc

Supplementary Fig. 2. Properties of the immortalized MEF lines.

a Heatmap showing the combinations of transgenes used to establish transformed MEFOG2 and ICR lines.

b Bright field views of the ICR and OG2-derived MEF lines. Scale bar = 100 μm.

c Heatmap of the expression for the transgenes used in this study in the OG2 and ICR-derived MEF lines.

d RT-qPCR for the indicated transgenes in the ICR derived MEF lines. Data is the mean of two biological replicates. X indicates not performed.

e Western blot for the indicated transgenes in the in the OG2 derived MEF lines. Beta-Actin was used as a control. The experiment was performed twice with similar results.

f Growth curves of the immortalized MEF ICR-lines (left chart), and estimated doubling time (right chart). Dots are the mean from three biological replicates. Bars indicate the standard deviation.

g Representative tumor images from MEFICR-derived growths in nude mice, images. Scale bar = 1cm. The experiment was performed twice with similar results.

h Chart of tumor sizes generated from the ICR MEFs from the indicated transformed lines. The middle bar represents the mean of the two biological replicates.

i Hematoxylin and eosin staining of cross sections of tumors generated from ICR MEFs transformed with the indicated transgenes. Scale bar = 100 μm. Two slides were prepared, and at least three views were taken per slide.

j Karyotype of the indicated ICR MEFs transformed with the indicated transgenes. This experiment was performed twice with similar results, and at least three karyotypes were inspected per biological replicate.

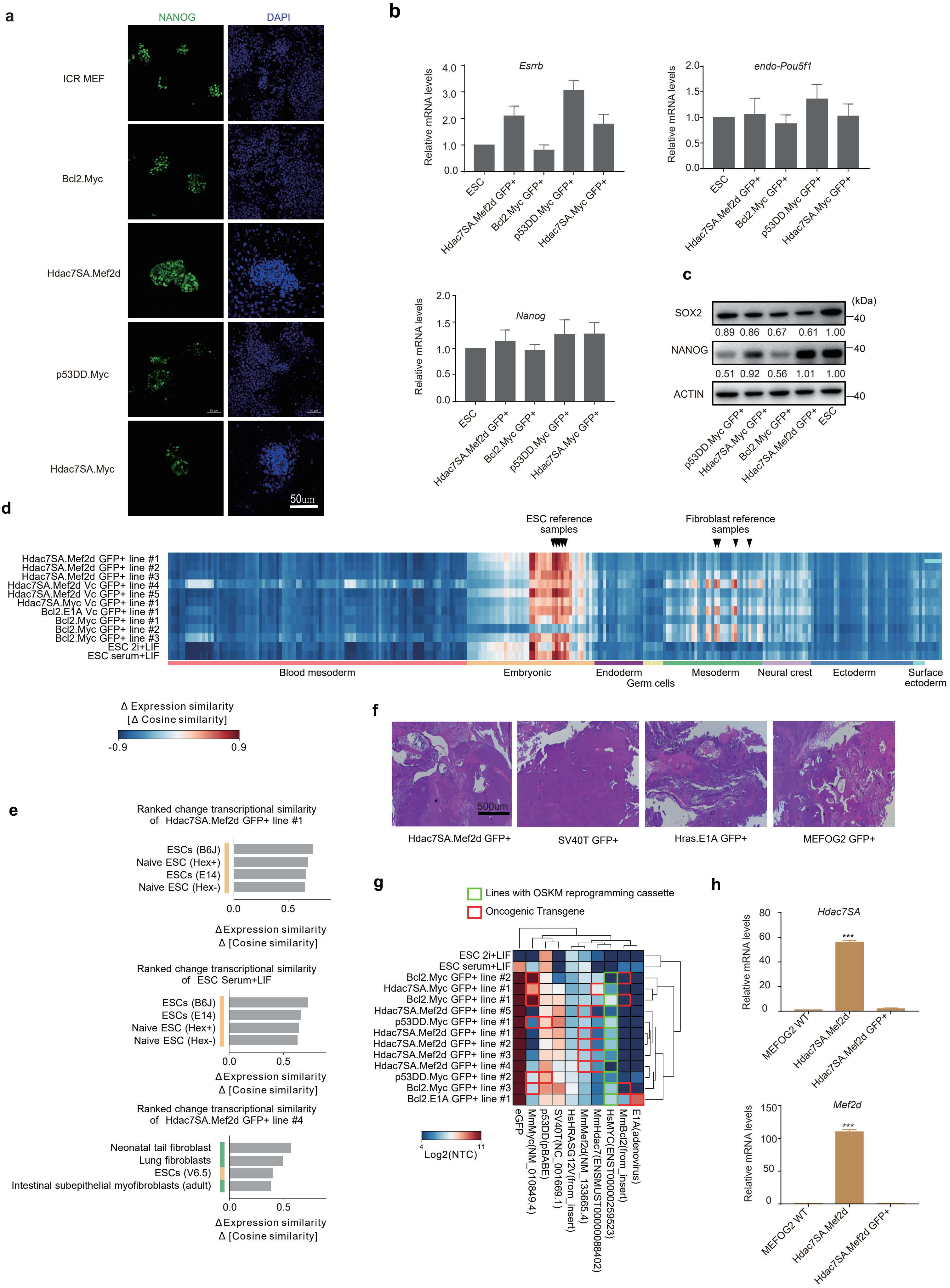
a
NANOG
DAPI
ICR MEF
Bcl2.Myc
Hdac7SA.Mef2d
p53DD.Myc
Hdac7SA.Myc
50um
b
Esrrb
Relative mRNA levels
4.0
3.0
2.0
1.0
0.0
ESC
Hdac7SA.Mef2d GFP+
Bcl2.Myc GFP+
p53DD.Myc GFP+
Hdac7SA.Myc GFP+
endo-Pou5f1
2.0
1.5
1.0
0.5
0.0
Nanog
c
SOX2
NANOG
ACTIN
(kDa)
40
0.89 0.86 0.67 0.61 1.00
0.51 0.92 0.56 1.01 1.00
p53DD.Myc GFP+
Hdac7SA.Myc GFP+
Bcl2.Myc GFP+
Hdac7SA.Mef2d GFP+
ESC
d
ESC reference samples
Fibroblast reference samples
Hdac7SA.Mef2d GFP+ line #1
Hdac7SA.Mef2d GFP+ line #2
Hdac7SA.Mef2d GFP+ line #3
Hdac7SA.Mef2d Vc GFP+ line #4
Hdac7SA.Mef2d Vc GFP+ line #5
Hdac7SA.Myc Vc GFP+ line #1
Bcl2.E1A Vc GFP+ line #1
Bcl2.Myc GFP+ line #1
Bcl2.Myc GFP+ line #2
Bcl2.Myc GFP+ line #3
ESC 2i+LIF
ESC serum+LIF
Blood mesoderm
Embryonic
Endoderm
Germ cells
Mesoderm
Neural crest
Ectoderm
Surface ectoderm
Δ Expression similarity
[Δ Cosine similarity]
-0.9
0.9
e
Ranked change transcriptional similarity of Hdac7SA.Mef2d GFP+ line #1
ESCs (B6J)
Naive ESC (Hex+)
ESCs (E14)
Naive ESC (Hex-)
0.0
0.5
ΔExpression similarity
Δ [Cosine similarity]
Ranked change transcriptional similarity of ESC Serum+LIF
ESCs (B6J)
ESCs (E14)
Naive ESC (Hex+)
Naive ESC (Hex-)
Ranked change transcriptional similarity of Hdac7SA.Mef2d GFP+ line #4
Neonatal tail fibroblast
Lung fibroblasts
ESCs (V6.5)
Intestinal subepithelial myofibroblasts (adult)
f
500um
Hdac7SA.Mef2d GFP+
SV40T GFP+
Hras.E1A GFP+
MEFOG2 GFP+
g
Lines with OSKM reprogramming cassette
Oncogenic Transgene
ESC 2i+LIF
ESC serum+LIF
Bcl2.Myc GFP+ line #2
Hdac7SA.Myc GFP+ line #1
Bcl2.Myc GFP+ line #1
Hdac7SA.Mef2d GFP+ line #5
p53DD.Myc GFP+ line #1
Hdac7SA.Mef2d GFP+ line #1
Hdac7SA.Mef2d GFP+ line #2
Hdac7SA.Mef2d GFP+ line #3
Hdac7SA.Mef2d GFP+ line #4
p53DD.Myc GFP+ line #2
Bcl2.Myc GFP+ line #3
Bcl2.E1A GFP+ line #1
eGFP
MmMyc(NM_010849.4)
p53DD(pBABE)
SV40T(NC_001669.1)
HsHRASG12V(from_insert)
MmMef2d(NM_133665.4)
MmHdac7(ENSMUST00000088402)
HsMYC(ENST00000259523)
MmBcl2(from_insert)
E1A(adenovirus)
4
11
Log2(NTC)
h
Hdac7SA
Relative mRNA levels
80
60
40
20
0
***
MEFOG2 WT
Hdac7SA.Mef2d
Hdac7SA.Mef2d GFP+
Mef2d
150
100
50
0
***

Supplementary Fig. 3. Reprogramming status of the iPSC lines derived from the immortalized MEFs.

- **a** Fluorescent microscopy images for NANOG for reprogramming of the indicated lines of the transformed ICR MEFs. Scale bar=50 μm. Images are representative and are from two biological replicates with three images per slide.
- **b** RT-qPCR for a selection of pluripotency-related genes in the GFP+ cells at day 15. RT-qPCR was performed our times from independent experiments.
- **c** Western blot for SOX2, NANOG and ACTIN (control) in the ESCs and the indicated transformed MEF lines. Experiment was performed twice with similar results.
- **d** Correlation of the iPSCs versus a large panel of mouse RNA-seq data (Hutchins et al., 2017), using DPre (Steffens et al., 2020).
- **e** Expression similarity as determined by DPre for the Hdac7SA.Mef2d, and ESC lines.
- **f** Representative images from hematoxylin and eosin-stained teratomas sections from the indicated transformed MEF lines.
- **g** RNA-seq data for the transgenes used in this study. (See also Figure S12c). The reprogramming OSKM cassette is a polycistronic vector but only the HsMYC gene is detectable in the RNA-seq data. Note that the p53DD is hard to determine by RNA-seq as the endogenous p53 interferes with the exogenous transgene. Oncogenic transgenes used to generate the MEF lines are indicated in red, and those cells with the OSKM cassette are marked in green.
- **h** RT-qPCR for *Hdac7SA* and *Mef2d* transgenes in the GFP+ iPSC-like lines. The mean of three biological replicates ± s.d. is shown. ***$P < 0.01$.

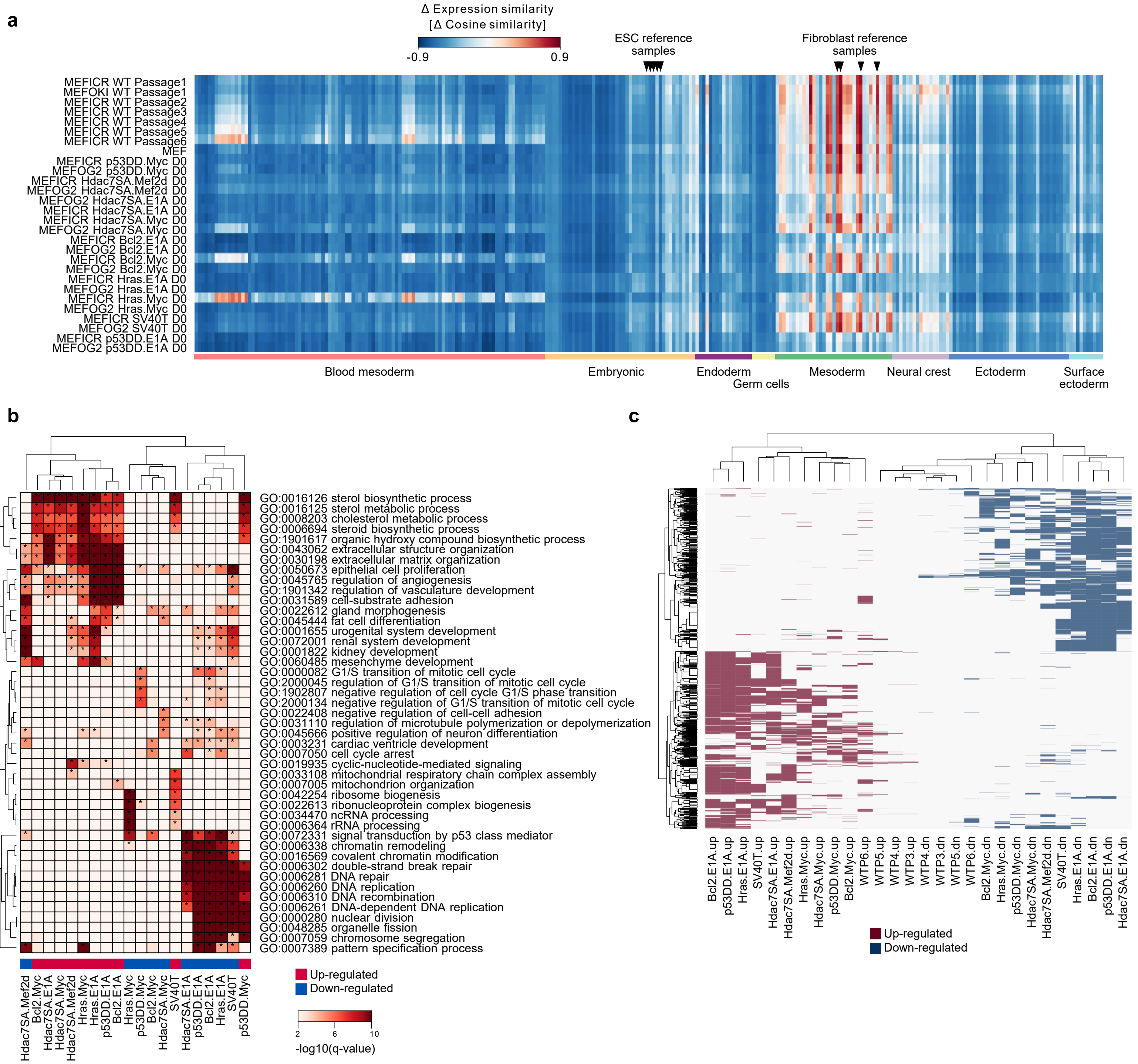
a
Δ Expression similarity
[Δ Cosine similarity]
-0.9
0.9
ESC reference samples
Fibroblast reference samples
MEFICR WT Passage1
MEFOKI WT Passage1
MEFICR WT Passage2
MEFICR WT Passage3
MEFICR WT Passage4
MEFICR WT Passage5
MEFICR WT Passage6
MEF
MEFICR p53DD.Myc D0
MEFOG2 p53DD.Myc D0
MEFICR Hdac7SA.Mef2d D0
MEFOG2 Hdac7SA.Mef2d D0
MEFOG2 Hdac7SA.E1A D0
MEFICR Hdac7SA.E1A D0
MEFICR Hdac7SA.Myc D0
MEFOG2 Hdac7SA.Myc D0
MEFICR Bcl2.E1A D0
MEFOG2 Bcl2.E1A D0
MEFICR Bcl2.Myc D0
MEFOG2 Bcl2.Myc D0
MEFICR Hras.E1A D0
MEFOG2 Hras.E1A D0
MEFICR Hras.Myc D0
MEFOG2 Hras.Myc D0
MEFICR SV40T D0
MEFOG2 SV40T D0
MEFICR p53DD.E1A D0
MEFOG2 p53DD.E1A D0
Blood mesoderm
Embryonic
Endoderm
Germ cells
Mesoderm
Neural crest
Ectoderm
Surface ectoderm
b
GO:0016126 sterol biosynthetic process
GO:0016125 sterol metabolic process
GO:0008203 cholesterol metabolic process
GO:0006694 steroid biosynthetic process
GO:1901617 organic hydroxy compound biosynthetic process
GO:0043062 extracellular structure organization
GO:0030198 extracellular matrix organization
GO:0050673 epithelial cell proliferation
GO:0045765 regulation of angiogenesis
GO:1901342 regulation of vasculature development
GO:0031589 cell-substrate adhesion
GO:0022612 gland morphogenesis
GO:0045444 fat cell differentiation
GO:0001655 urogenital system development
GO:0072001 renal system development
GO:0001822 kidney development
GO:0060485 mesenchyme development
GO:0000082 G1/S transition of mitotic cell cycle
GO:2000045 regulation of G1/S transition of mitotic cell cycle
GO:1902807 negative regulation of cell cycle G1/S phase transition
GO:2000134 negative regulation of G1/S transition of mitotic cell cycle
GO:0022408 negative regulation of cell-cell adhesion
GO:0031110 regulation of microtubule polymerization or depolymerization
GO:0045666 positive regulation of neuron differentiation
GO:0003231 cardiac ventricle development
GO:0007050 cell cycle arrest
GO:0019935 cyclic-nucleotide-mediated signaling
GO:0033108 mitochondrial respiratory chain complex assembly
GO:0007005 mitochondrion organization
GO:0042254 ribosome biogenesis
GO:0022613 ribonucleoprotein complex biogenesis
GO:0034470 ncRNA processing
GO:0006364 rRNA processing
GO:0072331 signal transduction by p53 class mediator
GO:0006338 chromatin remodeling
GO:0016569 covalent chromatin modification
GO:0006302 double-strand break repair
GO:0006281 DNA repair
GO:0006260 DNA replication
GO:0006310 DNA recombination
GO:0006261 DNA-dependent DNA replication
GO:0000280 nuclear division
GO:0048285 organelle fission
GO:0007059 chromosome segregation
GO:0007389 pattern specification process
Hdac7SA.Mef2d
Bcl2.Myc
Hdac7SA.E1A
Hdac7SA.Myc
Hdac7SA.Mef2d
Hras.Myc
Hras.E1A
p53DD.E1A
Bcl2.E1A
Hras.Myc
p53DD.Myc
Bcl2.Myc
Hdac7SA.Myc
SV40T
Hdac7SA.E1A
p53DD.E1A
Bcl2.E1A
Hras.E1A
SV40T
p53DD.Myc
Up-regulated
Down-regulated
2
6
10
-log10(q-value)
c
Bcl2.E1A.up
p53DD.E1A.up
Hras.E1A.up
SV40T.up
Hdac7SA.E1A.up
Hdac7SA.Mef2d.up
Hras.Myc.up
Hdac7SA.Myc.up
p53DD.Myc.up
Bcl2.Myc.up
WTP6.up
WTP5.up
WTP4.up
WTP3.up
WTP4.dn
WTP3.dn
WTP5.dn
WTP6.dn
Bcl2.Myc.dn
Hras.Myc.dn
p53DD.Myc.dn
Hdac7SA.Myc.dn
Hdac7SA.Mef2d.dn
SV40T.dn
Hras.E1A.dn
Bcl2.E1A.dn
p53DD.E1A.dn
Hdac7SA.E1A.dn
Up-regulated
Down-regulated

Supplementary Fig. 4. Gene expression and epigenetic changes in the transformed immortal MEF lines.

- **a** No evidence that the MEFs are differentiating, correlations versus the closest samples from (Hutchins et al., 2017) using DPre (Steffens et al., 2020).
- **b** Gene ontology analysis of the DE genes in each MEF line. Up-regulated genes are in the left heatmap and down-regulated genes are in the right heatmap.
- **c** Heatmap of significantly up (red) and down-regulated (blue) genes in the indicated MEF transformed cell lines. Heatmap was generated using any genes that appeared in any four cell lines (either up or down-regulated).

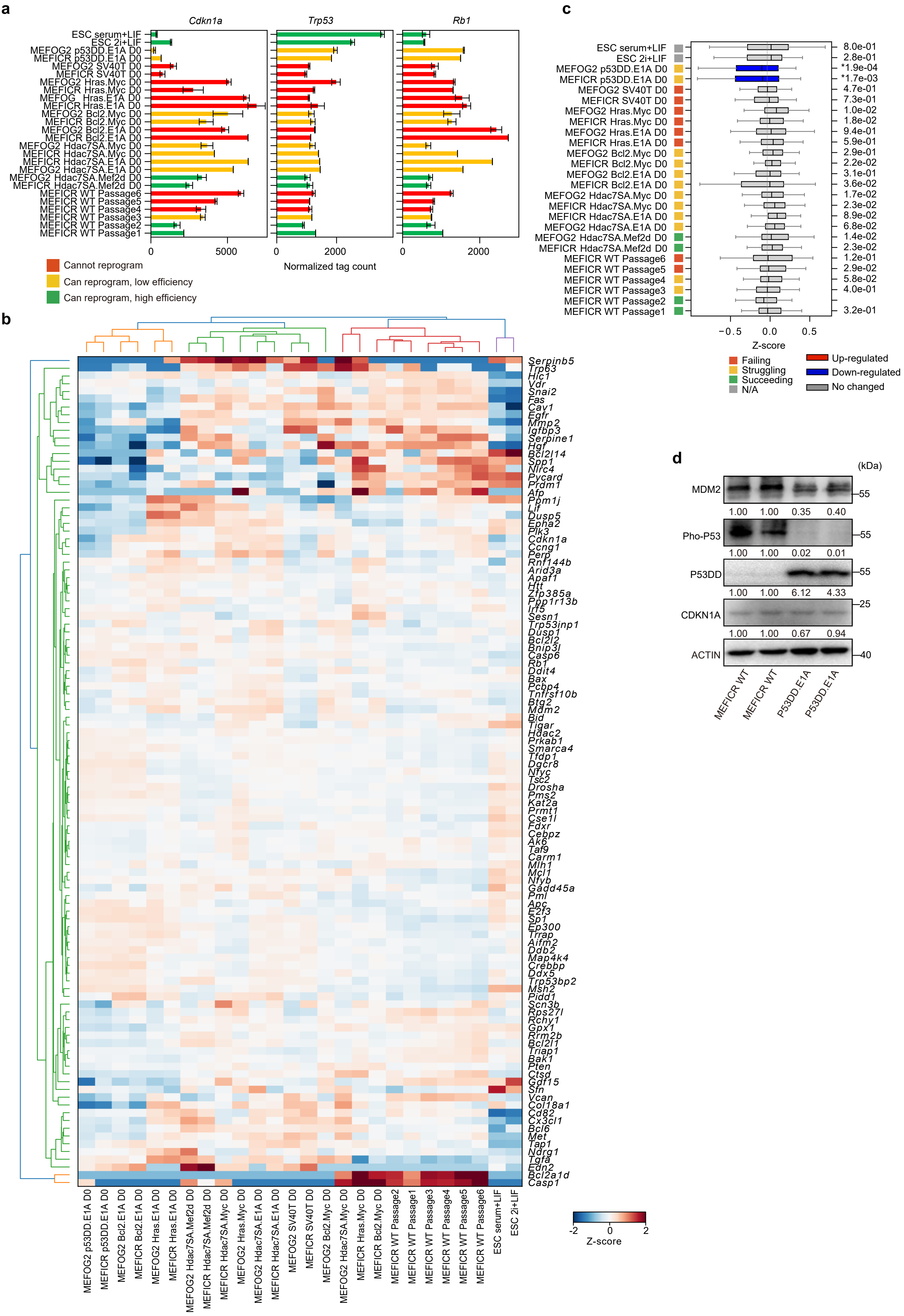

a
Cdkn1a
Trp53
Rb1
ESC serum+LIF
ESC 2i+LIF
MEFOG2 p53DD.E1A D0
MEFICR p53DD.E1A D0
MEFOG2 SV40T D0
MEFICR SV40T D0
MEFOG2 Hras.Myc D0
MEFICR Hras.Myc D0
MEFOG Hras.E1A D0
MEFICR Hras.E1A D0
MEFOG2 Bcl2.Myc D0
MEFICR Bcl2.Myc D0
MEFOG2 Bcl2.E1A D0
MEFICR Bcl2.E1A D0
MEFOG2 Hdac7SA.Myc D0
MEFICR Hdac7SA.Myc D0
MEFICR Hdac7SA.E1A D0
MEFOG2 Hdac7SA.E1A D0
MEFOG2 Hdac7SA.Mef2d D0
MEFICR Hdac7SA.Mef2d D0
MEFICR WT Passage6
MEFICR WT Passage5
MEFICR WT Passage4
MEFICR WT Passage3
MEFICR WT Passage2
MEFICR WT Passage1
0
5000
0
2000
0
2000
Normalized tag count
Cannot reprogram
Can reprogram, low efficiency
Can reprogram, high efficiency
b
Serpinb5
Trp63
Hic1
Vdr
Snai2
Fas
Cav1
Egfr
Mmp2
Igfbp3
Serpine1
Hgf
Bcl2l14
Spp1
Nlrc4
Pycard
Prdm1
Afp
Ppm1j
Lif
Dusp5
Epha2
Plk3
Cdkn1a
Ccng1
Perp
Rnf144b
Arid3a
Apaf1
Htt
Zfp385a
Ppp1r13b
Irf5
Sesn1
Trp53inp1
Dusp1
Bcl2l2
Bnip3l
Casp6
Rb1
Ddit4
Bax
Pcbp4
Tnfrsf10b
Btg2
Mdm2
Bid
Tigar
Hdac2
Prkab1
Smarca4
Tfdp1
Dgcr8
Nfyc
Tsc2
Drosha
Pms2
Kat2a
Prmt1
Cse1l
Fdxr
Cebpz
Ak6
Taf9
Carm1
Mlh1
Mcl1
Nfyb
Gadd45a
Pml
Apc
E2f3
Sp1
Ep300
Trrap
Aifm2
Ddb2
Map4k4
Crebbp
Ddx5
Trp53bp2
Msh2
Pidd1
Scn3b
Rps27l
Rchy1
Gpx1
Rrm2b
Bcl2l1
Triap1
Bak1
Pten
Ctsd
Gdf15
Sfn
Vcan
Col18a1
Cd82
Cx3cl1
Bcl6
Met
Tap1
Ndrg1
Tgfa
Edn2
Bcl2a1d
Casp1
MEFOG2 p53DD.E1A D0
MEFICR p53DD.E1A D0
MEFOG2 Bcl2.E1A D0
MEFICR Bcl2.E1A D0
MEFOG2 Hras.E1A D0
MEFICR Hras.E1A D0
MEFOG2 Hdac7SA.Mef2d D0
MEFICR Hdac7SA.Mef2d D0
MEFICR Hdac7SA.Myc D0
MEFOG2 Hras.Myc D0
MEFOG2 Hdac7SA.E1A D0
MEFICR Hdac7SA.E1A D0
MEFOG2 SV40T D0
MEFICR SV40T D0
MEFOG2 Bcl2.Myc D0
MEFOG2 Hdac7SA.Myc D0
MEFICR Hras.Myc D0
MEFICR Bcl2.Myc D0
MEFICR WT Passage2
MEFICR WT Passage1
MEFICR WT Passage3
MEFICR WT Passage4
MEFICR WT Passage5
MEFICR WT Passage6
ESC serum+LIF
ESC 2i+LIF
-2
0
2
Z-score
c
ESC serum+LIF
ESC 2i+LIF
MEFOG2 p53DD.E1A D0
MEFICR p53DD.E1A D0
MEFOG2 SV40T D0
MEFICR SV40T D0
MEFOG2 Hras.Myc D0
MEFICR Hras.Myc D0
MEFOG2 Hras.E1A D0
MEFICR Hras.E1A D0
MEFOG2 Bcl2.Myc D0
MEFICR Bcl2.Myc D0
MEFOG2 Bcl2.E1A D0
MEFICR Bcl2.E1A D0
MEFOG2 Hdac7SA.Myc D0
MEFICR Hdac7SA.Myc D0
MEFICR Hdac7SA.E1A D0
MEFOG2 Hdac7SA.E1A D0
MEFOG2 Hdac7SA.Mef2d D0
MEFICR Hdac7SA.Mef2d D0
MEFICR WT Passage6
MEFICR WT Passage5
MEFICR WT Passage4
MEFICR WT Passage3
MEFICR WT Passage2
MEFICR WT Passage1
8.0e-01
2.8e-01
*1.9e-04
*1.7e-03
4.7e-01
7.3e-01
1.0e-02
1.8e-02
9.4e-01
5.9e-01
2.9e-01
2.2e-02
3.1e-01
3.6e-02
1.7e-02
2.3e-02
8.9e-02
6.8e-02
1.4e-02
2.3e-02
1.2e-01
2.9e-02
5.8e-02
4.0e-01
3.2e-01
−0.5
0.0
0.5
Z-score
Failing
Struggling
Succeeding
N/A
Up-regulated
Down-regulated
No changed
d
(kDa)
MDM2
–55
1.00
1.00
0.35
0.40
Pho-P53
–55
1.00
1.00
0.02
0.01
P53DD
–55
1.00
1.00
6.12
4.33
–25
CDKN1A
1.00
1.00
0.67
0.94
ACTIN
–40
MEFICR WT
MEFICR WT
P53DD.E1A
P53DD.E1A

Supplementary Fig. 5. Known blocks on reprogramming are not responsible for the inability of transformed MEFs to reprogram

- **a** Bar chart of the expression of *Tp53*, *Cdkn1a* and *Rb1* expression in transformed MEF lines and the first 6 passages of wildtype MEFs
- **b** Z-score heatmap of p53 target genes (taken from PID_P53_DOWNSTREAM_PATHWAY MSIGDB_C2)
- **c** Box plots of the genes in **panel b**, significance is versus MEFICR WT Passage 2 samples, calculated from a two-sided Welch's t-test. Blue indicates significantly down-regulated.
- **d** Western blot for MDM2, phosphorylated-p53 (Pho-p53), p53DD, CDKN1A or ACTIN in ICR MEFs and ICR MEFs transformed with p53DD.E1A transgenes. Beta-Actin was used as a control. This experiment was performed twice with similar results.

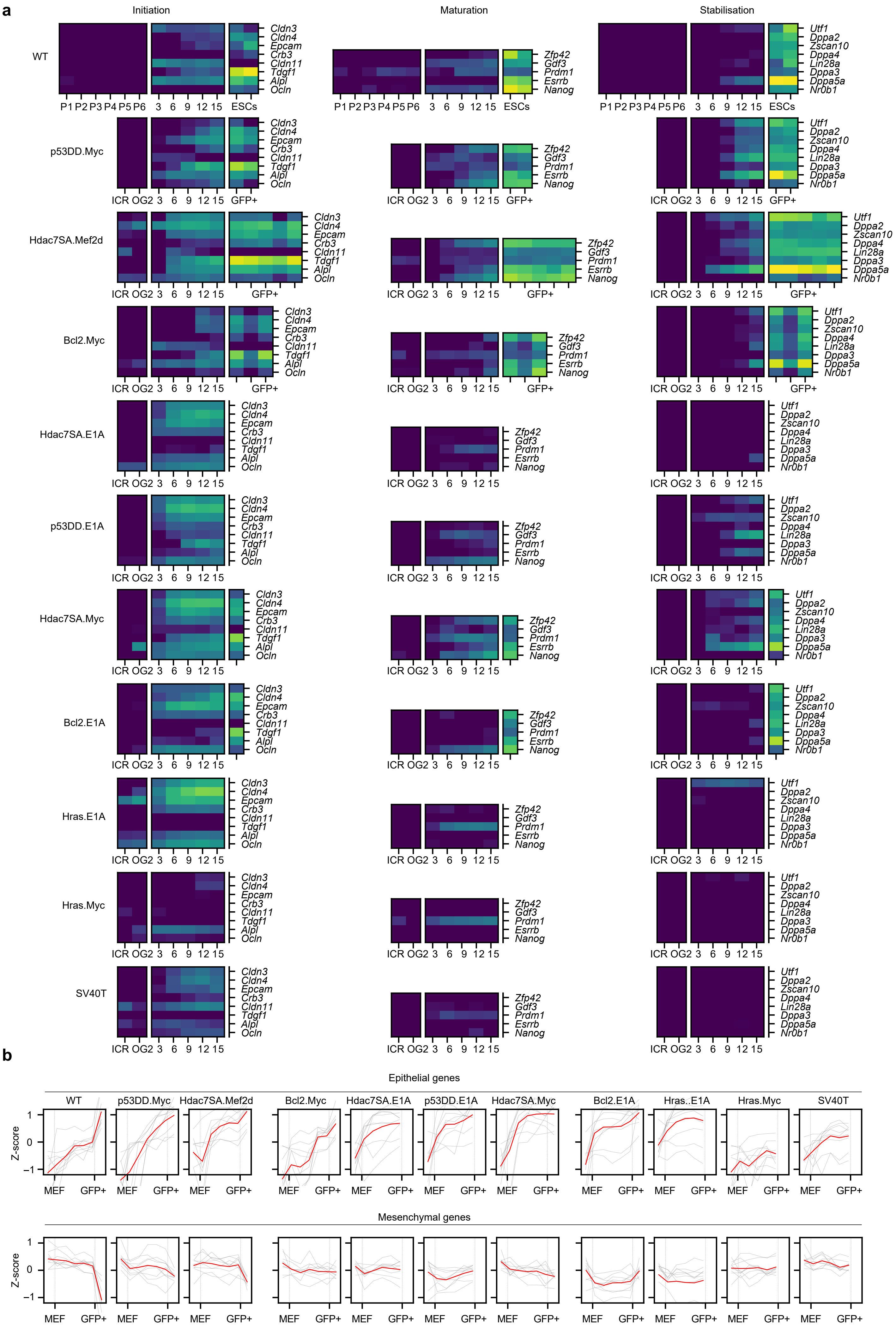
a
Initiation
Maturation
Stabilisation
WT
p53DD.Myc
Hdac7SA.Mef2d
Bcl2.Myc
Hdac7SA.E1A
p53DD.E1A
Hdac7SA.Myc
Bcl2.E1A
Hras.E1A
Hras.Myc
SV40T
P1 P2 P3 P4 P5 P6 3 6 9 12 15 ESCs
ICR OG2 3 6 9 12 15 GFP+
Cldn3
Cldn4
Epcam
Crb3
Cldn11
Tdgf1
Alpl
Ocln
Zfp42
Gdf3
Prdm1
Esrrb
Nanog
Utf1
Dppa2
Zscan10
Dppa4
Lin28a
Dppa3
Dppa5a
Nr0b1
b
Epithelial genes
Mesenchymal genes
WT
p53DD.Myc
Hdac7SA.Mef2d
Bcl2.Myc
Hdac7SA.E1A
p53DD.E1A
Hdac7SA.Myc
Bcl2.E1A
Hras..E1A
Hras.Myc
SV40T
Z-score
1
0
−1
MEF
GFP+

Supplementary Fig. 6. Transformed cells have disrupted phases of reprogramming

a Heatmaps showing the expression of initiation (left panel), maturation (middle panel) and stabilization (right panel) genes (Samavarchi-Tehrani et al., 2010), at the indicated time points (days) or in ICR/OG2 MEFs, ESCs, and GFP+ cell lines.

b Line charts showing the expression of mesenchymal and epithelial genes at the indicated time points or in ICR/OG2 MEFs and GFP+ cells. The red line is the mean of all genes, the grey lines are the individual genes.

a

Drug name	Function	Effect
PD0325901	ERK/MEK inhibitor	Promote
CHIR99021	GSK inhibitor	Promote
Y23637	ROCK inhibitor	Promote
BIX-01294	Methyltransferase G9a inhibitor	Promote
TSA	HDAC inhibitor	Promote
Rapamycin	mTOR inhibitor	No effect
DZNep	Methyltransferase inhibitor	No effect
MS023	Methyltransferase Pan-PRMT inhibitor	No effect
OICR9429	Methyltransferase WDR-family inhibitor	No effect
Chaetocin	Methyltransferase Suv39h1/2 inhibitor	No effect
UNC0379	Methyltransferase SETD8 inhibitor	No effect
CPTH2	HAT inhibitor	No effect
Garcinol	HAT inhibitor	No effect
Sirtinol	Deacetylation SIRT1/2 inhibitor	No effect
JQ1	BRD4 inhibitor	No effect
Bromosporine	Bromodomain Pan-BR inhibitor	No effect
AZD6738	Ras inhibitor	No effect
BMH-21	DNA/RNA synthesis inhibitor	No effect
CX5461	DNA/RNA synthesis inhibitor	No effect
BX-795	PDK1 inhibitor	No effect
GW843682X	Cell cycle inhibitor	No effect
BX-912	PDK1 inhibitor	No effect
10058-F4	Cell cycle inhibitor	No effect
ICP-203	Cell cycle inhibitor	No effect
Flavopiridol	CDK inhibitor	No effect

b

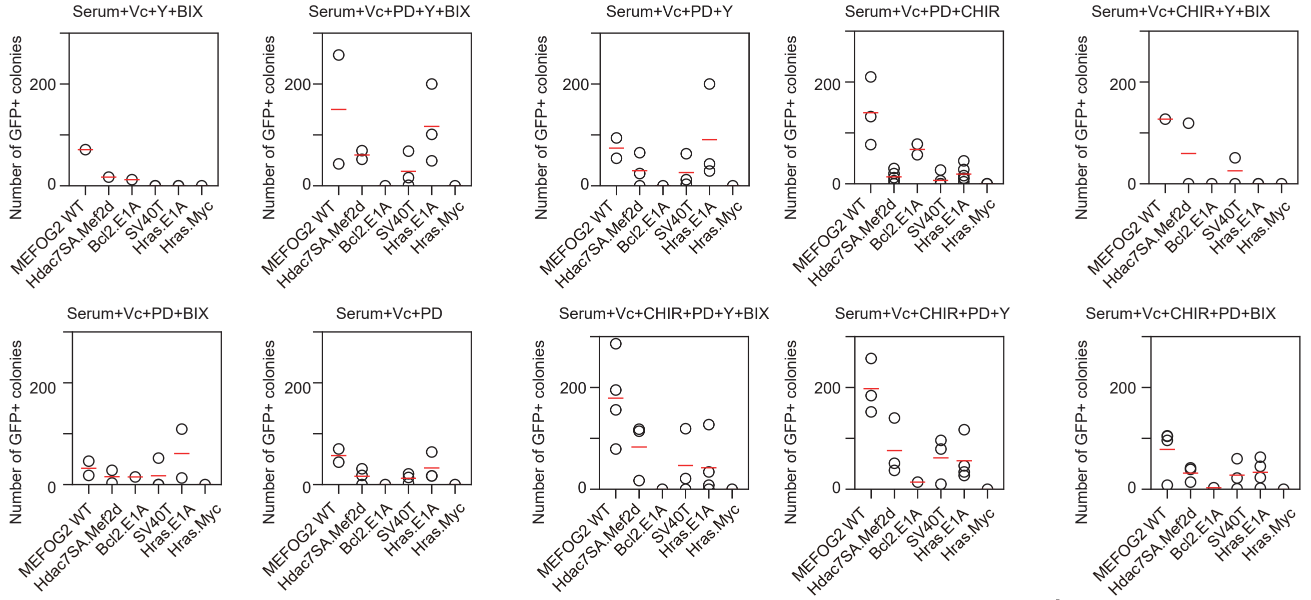
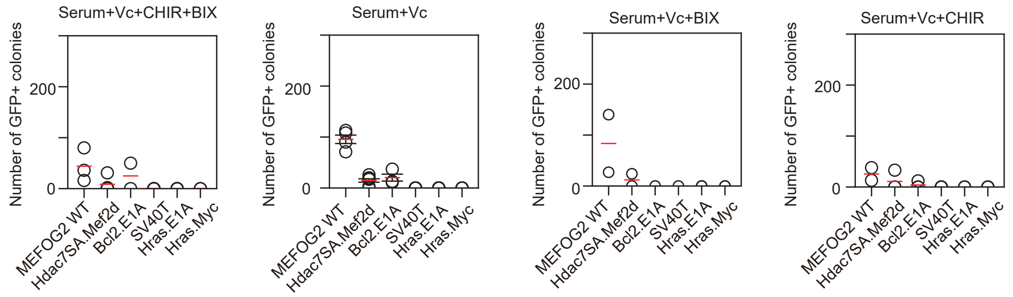

c

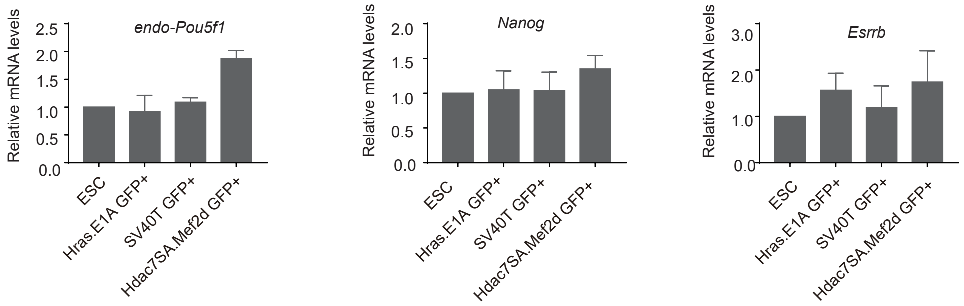

d

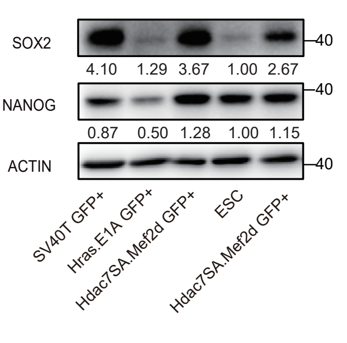

Supplementary Fig. 7. Development of the inhibitor cocktail to convert reprogramming-incapable cells to -capable.

a A summary of the inhibitors used in this study.

b GFP+ colony counts from reprogramming experiments for the indicated inhibitor cocktail combinations. Data is from a varied number of biological replicates per transformed cell line and inhibitor treatment. Experiments were performed twice with two biological replicates (Serum+Vc+PD+Y+BIX, Serum+Vc+PD+Y, Serum+Vc+CHIR+Y+BIX, Serum+Vc+PD+BIX, Serum+Vc+PD, Serum+Vc+BIX, Serum+Vc+CHIR) or in triplicate (Serum+Vc+PD+CHIR, Serum+Vc+PD+CHIR+Y, Serum+Vc+PD+CHIR+BIX, Serum+Vc+CHIR+BIX), or in quadruplicate (Serum+Vc+PD+CHIR+Y+BIX, Serum+Vc+PD+CHIR+BIX, serum+Vc). One experiment was not replicated (Serum+Y+BIX).

c RT-qPCR at day 15 of a reprogramming experiment using primers targeting endogenous Oct4 (endo-*Pou5f1*), *Nanog, and Esrrb*. The mean of three biological replicates ± s.d. is shown.

d Western blot for NANOG, SOX2 and ACTB as a loading control in the indicated GFP+ lines derived from the indicated WT or transformed MEFs. Experiment was performed twice with similar results.

a

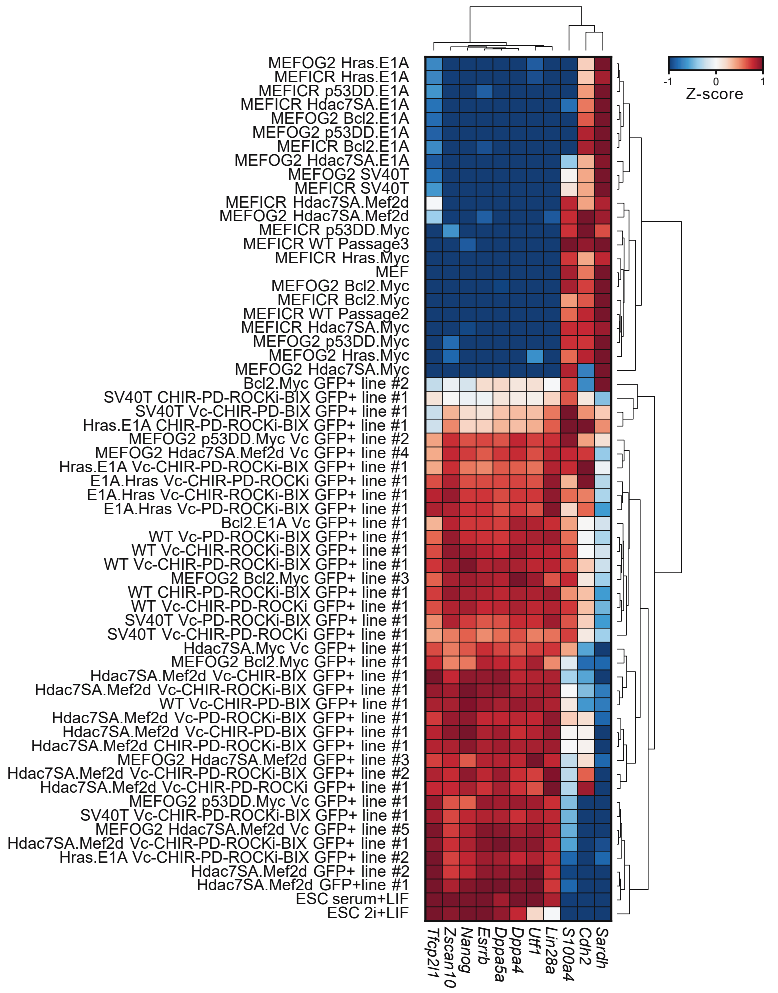

MEFOG2 Hras.E1A
MEFICR Hras.E1A
MEFICR p53DD.E1A
MEFICR Hdac7SA.E1A
MEFOG2 Bcl2.E1A
MEFOG2 p53DD.E1A
MEFICR Bcl2.E1A
MEFOG2 Hdac7SA.E1A
MEFOG2 SV40T
MEFICR SV40T
MEFICR Hdac7SA.Mef2d
MEFOG2 Hdac7SA.Mef2d
MEFICR p53DD.Myc
MEFICR WT Passage3
MEFICR Hras.Myc
MEF
MEFOG2 Bcl2.Myc
MEFICR Bcl2.Myc
MEFICR WT Passage2
MEFICR Hdac7SA.Myc
MEFOG2 p53DD.Myc
MEFOG2 Hras.Myc
MEFOG2 Hdac7SA.Myc
Bcl2.Myc GFP+ line #2
SV40T CHIR-PD-ROCKi-BIX GFP+ line #1
SV40T Vc-CHIR-PD-BIX GFP+ line #1
Hras.E1A CHIR-PD-ROCKi-BIX GFP+ line #1
MEFOG2 p53DD.Myc Vc GFP+ line #2
MEFOG2 Hdac7SA.Mef2d Vc GFP+ line #4
Hras.E1A Vc-CHIR-PD-ROCKi-BIX GFP+ line #1
E1A.Hras Vc-CHIR-PD-ROCKi GFP+ line #1
E1A.Hras Vc-CHIR-ROCKi-BIX GFP+ line #1
E1A.Hras Vc-PD-ROCKi-BIX GFP+ line #1
Bcl2.E1A Vc GFP+ line #1
WT Vc-PD-ROCKi-BIX GFP+ line #1
WT Vc-CHIR-ROCKi-BIX GFP+ line #1
WT Vc-CHIR-PD-ROCKi-BIX GFP+ line #1
MEFOG2 Bcl2.Myc GFP+ line #3
WT CHIR-PD-ROCKi-BIX GFP+ line #1
WT Vc-CHIR-PD-ROCKi GFP+ line #1
SV40T Vc-PD-ROCKi-BIX GFP+ line #1
SV40T Vc-CHIR-PD-ROCKi GFP+ line #1
Hdac7SA.Myc Vc GFP+ line #1
MEFOG2 Bcl2.Myc GFP+ line #1
Hdac7SA.Mef2d Vc-CHIR-BIX GFP+ line #1
Hdac7SA.Mef2d Vc-CHIR-ROCKi-BIX GFP+ line #1
WT Vc-CHIR-PD-BIX GFP+ line #1
Hdac7SA.Mef2d Vc-PD-ROCKi-BIX GFP+ line #1
Hdac7SA.Mef2d Vc-CHIR-PD-BIX GFP+ line #1
Hdac7SA.Mef2d CHIR-PD-ROCKi-BIX GFP+ line #1
MEFOG2 Hdac7SA.Mef2d GFP+ line #3
Hdac7SA.Mef2d Vc-CHIR-PD-ROCKi-BIX GFP+ line #2
Hdac7SA.Mef2d Vc-CHIR-PD-ROCKi GFP+ line #1
MEFOG2 p53DD.Myc Vc GFP+ line #1
SV40T Vc-CHIR-PD-ROCKi-BIX GFP+ line #1
MEFOG2 Hdac7SA.Mef2d Vc GFP+ line #5
Hdac7SA.Mef2d Vc-CHIR-PD-ROCKi-BIX GFP+ line #1
Hras.E1A Vc-CHIR-PD-ROCKi-BIX GFP+ line #2
Hdac7SA.Mef2d GFP+ line #2
Hdac7SA.Mef2d GFP+line #1
ESC serum+LIF
ESC 2i+LIF
Tfcp2l1
Zscan10
Nanog
Esrrb
Dppa5a
Dppa4
Utf1
Lin28a
S100a4
Cdh2
Sardh
-1
0
1
Z-score

b

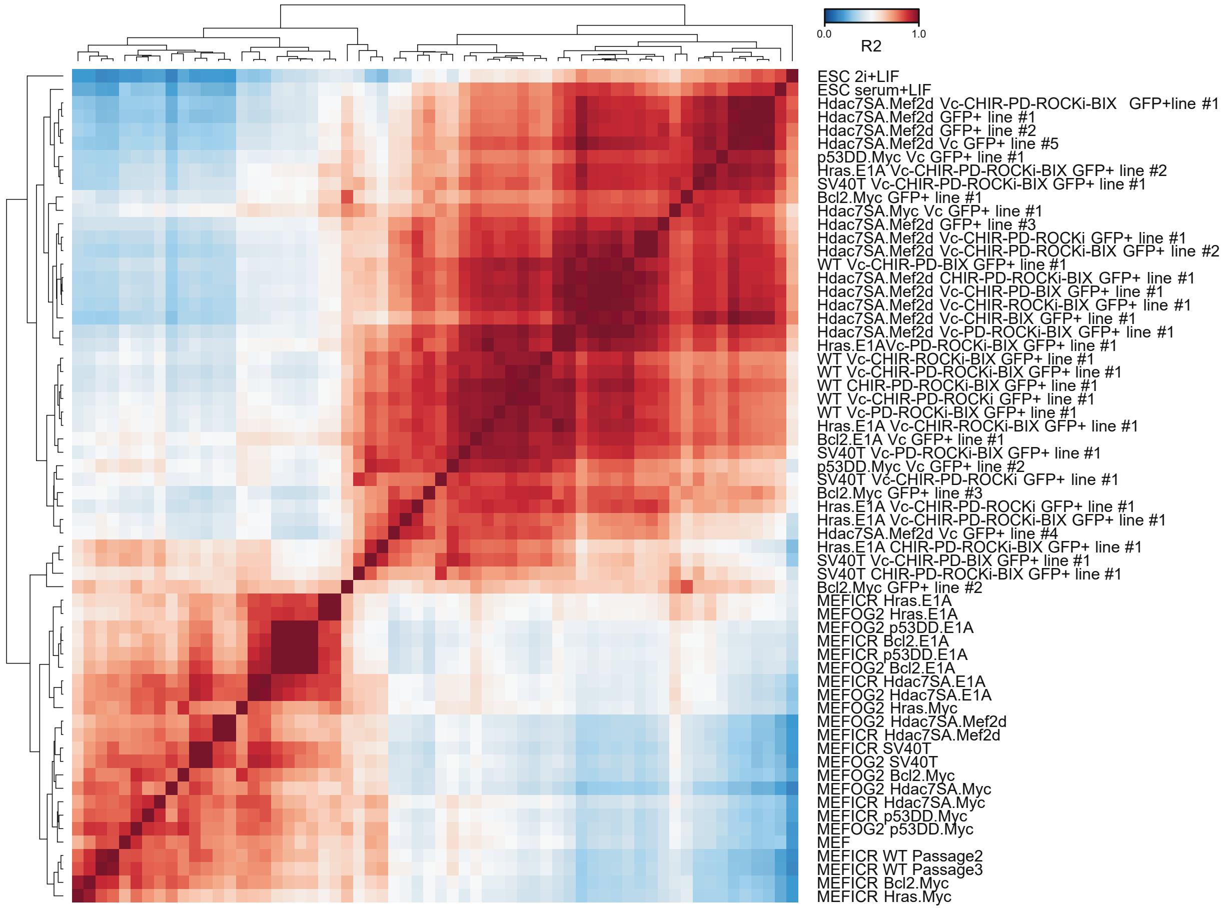

0.0
1.0
R2
ESC 2i+LIF
ESC serum+LIF
Hdac7SA.Mef2d Vc-CHIR-PD-ROCKi-BIX GFP+line #1
Hdac7SA.Mef2d GFP+ line #1
Hdac7SA.Mef2d GFP+ line #2
Hdac7SA.Mef2d Vc GFP+ line #5
p53DD.Myc Vc GFP+ line #1
Hras.E1A Vc-CHIR-PD-ROCKi-BIX GFP+ line #2
SV40T Vc-CHIR-PD-ROCKi-BIX GFP+ line #1
Bcl2.Myc GFP+ line #1
Hdac7SA.Myc Vc GFP+ line #1
Hdac7SA.Mef2d GFP+ line #3
Hdac7SA.Mef2d Vc-CHIR-PD-ROCKi GFP+ line #1
Hdac7SA.Mef2d Vc-CHIR-PD-ROCKi-BIX GFP+ line #2
WT Vc-CHIR-PD-BIX GFP+ line #1
Hdac7SA.Mef2d CHIR-PD-ROCKi-BIX GFP+ line #1
Hdac7SA.Mef2d Vc-CHIR-PD-BIX GFP+ line #1
Hdac7SA.Mef2d Vc-CHIR-ROCKi-BIX GFP+ line #1
Hdac7SA.Mef2d Vc-CHIR-BIX GFP+ line #1
Hdac7SA.Mef2d Vc-PD-ROCKi-BIX GFP+ line #1
Hras.E1AVc-PD-ROCKi-BIX GFP+ line #1
WT Vc-CHIR-ROCKi-BIX GFP+ line #1
WT Vc-CHIR-PD-ROCKi-BIX GFP+ line #1
WT CHIR-PD-ROCKi-BIX GFP+ line #1
WT Vc-CHIR-PD-ROCKi GFP+ line #1
WT Vc-PD-ROCKi-BIX GFP+ line #1
Hras.E1A Vc-CHIR-ROCKi-BIX GFP+ line #1
Bcl2.E1A Vc GFP+ line #1
SV40T Vc-PD-ROCKi-BIX GFP+ line #1
p53DD.Myc Vc GFP+ line #2
SV40T Vc-CHIR-PD-ROCKi GFP+ line #1
Bcl2.Myc GFP+ line #3
Hras.E1A Vc-CHIR-PD-ROCKi GFP+ line #1
Hras.E1A Vc-CHIR-PD-ROCKi-BIX GFP+ line #1
Hdac7SA.Mef2d Vc GFP+ line #4
Hras.E1A CHIR-PD-ROCKi-BIX GFP+ line #1
SV40T Vc-CHIR-PD-BIX GFP+ line #1
SV40T CHIR-PD-ROCKi-BIX GFP+ line #1
Bcl2.Myc GFP+ line #2
MEFICR Hras.E1A
MEFOG2 Hras.E1A
MEFOG2 p53DD.E1A
MEFICR Bcl2.E1A
MEFICR p53DD.E1A
MEFOG2 Bcl2.E1A
MEFICR Hdac7SA.E1A
MEFOG2 Hdac7SA.E1A
MEFOG2 Hras.Myc
MEFOG2 Hdac7SA.Mef2d
MEFICR Hdac7SA.Mef2d
MEFICR SV40T
MEFOG2 SV40T
MEFOG2 Bcl2.Myc
MEFOG2 Hdac7SA.Myc
MEFICR Hdac7SA.Myc
MEFICR p53DD.Myc
MEFOG2 p53DD.Myc
MEF
MEFICR WT Passage2
MEFICR WT Passage3
MEFICR Bcl2.Myc
MEFICR Hras.Myc

Supplementary Fig. 8. Reprogrammed cell lines closely resemble wildtype ESCs

- **a** Heatmap of the expression of selected pluripotency and somatic cell factors in the WT MEFs, transformed MEF cells lines, WT ESCs and the GFP+ sorted cell lines derived from the transformed MEFs. Chemical conditions used in the reprogramming process are indicated for each sample. If not stated then reprogramming was performed in serum+Vitamin C.
- **b** Cross-correlation (R^2) of the RNA-seq data for the WT MEFs, transformed MEF cells lines, WT ESCs and the GFP+ sorted cell lines derived from the transformed MEFs.

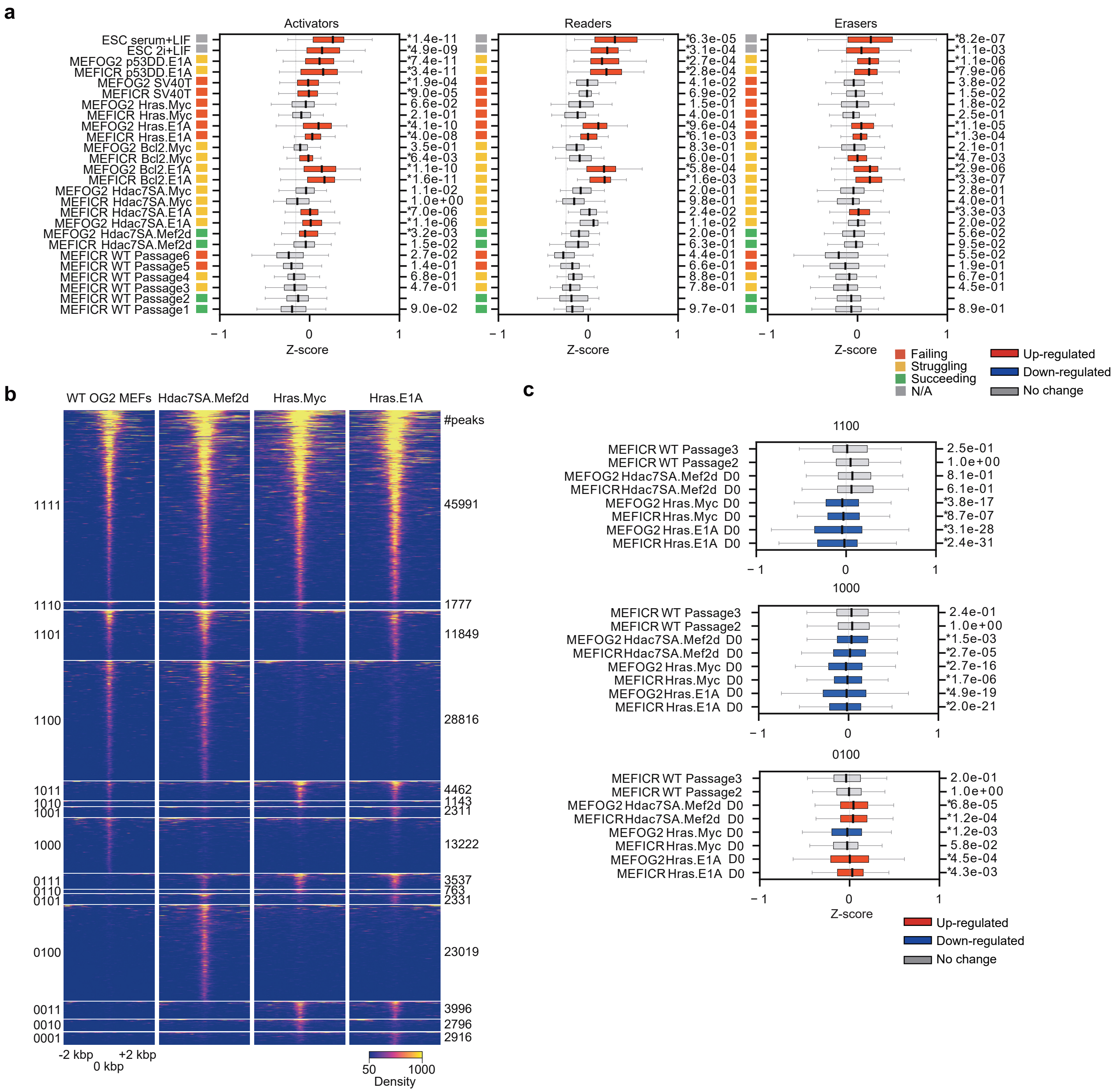
a
Activators
Readers
Erasers
ESC serum+LIF
ESC 2i+LIF
MEFOG2 p53DD.E1A
MEFICR p53DD.E1A
MEFOG2 SV40T
MEFICR SV40T
MEFOG2 Hras.Myc
MEFICR Hras.Myc
MEFOG2 Hras.E1A
MEFICR Hras.E1A
MEFOG2 Bcl2.Myc
MEFICR Bcl2.Myc
MEFOG2 Bcl2.E1A
MEFICR Bcl2.E1A
MEFOG2 Hdac7SA.Myc
MEFICR Hdac7SA.Myc
MEFICR Hdac7SA.E1A
MEFOG2 Hdac7SA.E1A
MEFOG2 Hdac7SA.Mef2d
MEFICR Hdac7SA.Mef2d
MEFICR WT Passage6
MEFICR WT Passage5
MEFICR WT Passage4
MEFICR WT Passage3
MEFICR WT Passage2
MEFICR WT Passage1
*1.4e-11
*4.9e-09
*7.4e-11
*3.4e-11
*1.9e-04
*9.0e-05
6.6e-02
2.1e-01
*4.1e-10
*4.0e-08
3.5e-01
*6.4e-03
*1.1e-10
*1.6e-11
1.1e-02
1.0e+00
*7.0e-06
*1.1e-06
*3.2e-03
1.5e-02
2.7e-02
1.4e-01
6.8e-01
4.7e-01
9.0e-02
*6.3e-05
*3.1e-04
*2.7e-04
*2.8e-04
4.1e-02
6.9e-02
1.5e-01
4.0e-01
*9.6e-04
*6.1e-03
8.3e-01
6.0e-01
*5.8e-04
*1.6e-03
2.0e-01
9.8e-01
2.4e-02
1.1e-02
2.0e-01
6.3e-01
4.4e-01
6.6e-01
8.8e-01
7.8e-01
9.7e-01
*8.2e-07
*1.1e-03
*1.1e-06
*7.9e-06
3.8e-02
1.5e-02
1.8e-02
2.5e-01
*1.1e-05
*1.3e-04
2.1e-01
*4.7e-03
*2.9e-06
*3.3e-07
2.8e-01
4.0e-01
*3.3e-03
2.0e-02
5.6e-02
9.5e-02
5.5e-02
1.9e-01
6.7e-01
4.5e-01
8.9e-01
– 1
0
1
Z-score
Failing
Struggling
Succeeding
N/A
Up-regulated
Down-regulated
No change
b
WT OG2 MEFs
Hdac7SA.Mef2d
Hras.Myc
Hras.E1A
#peaks
1111
45991
1110
1777
1101
11849
1100
28816
1011
4462
1010
1143
1001
2311
1000
13222
0111
3537
0110
763
0101
2331
0100
23019
0011
3996
0010
2796
0001
2916
-2 kbp
+2 kbp
0 kbp
50
1000
Density
c
1100
1000
0100
MEFICR WT Passage3
MEFICR WT Passage2
MEFOG2 Hdac7SA.Mef2d D0
MEFICR Hdac7SA.Mef2d D0
MEFOG2 Hras.Myc D0
MEFICR Hras.Myc D0
MEFOG2 Hras.E1A D0
MEFICR Hras.E1A D0
2.5e-01
1.0e+00
8.1e-01
6.1e-01
*3.8e-17
*8.7e-07
*3.1e-28
*2.4e-31
2.4e-01
1.0e+00
*1.5e-03
*2.7e-05
*2.7e-16
*1.7e-06
*4.9e-19
*2.0e-21
2.0e-01
1.0e+00
*6.8e-05
*1.2e-04
*1.2e-03
5.8e-02
*4.5e-04
*4.3e-03
– 1
0
1
Z-score
Up-regulated
Down-regulated
No change

Supplementary Fig. 9. Epigenetic control of reprogramming-capable and -incapable lines

- **a** Box plots of the expression of epigenetic factors in MEFs. Epigenetic factors were downloaded from the Epifactors database (Medvedeva et al., 2015), and allocated to one of three categories: Activator, Reader or Eraser. The Z-scores of expression is indicated. Significance is from a Welch's test with MEFICR Passage2 compared to all other samples. Changes were considered significant if the p-value was < 0.01.
- **b** Heatmap comparing the ATAC-seq data in WT MEFs to the ATAC-seq in the indicated transformed cell lines, for the indicated binary classifications of open (1) or not open (0) shown on the left side of the heatmap. The plot is centered on the peak summit and shows the flanking 1 kbp. Normalized tag counts are shown for the indicated samples.
- **c** Boxplots of the expression of all genes within 2 kbp of a set of loci that are specifically opened or closed in the indicated lines. Three groups of peaks are shown, 1100 (open in WT MEFs and Hdac7SA.Mef2d, closed in the other lines), 1000 (only open in WT MEFs), and 0100 (specifically open in the Hdac7SA.Mef2d).

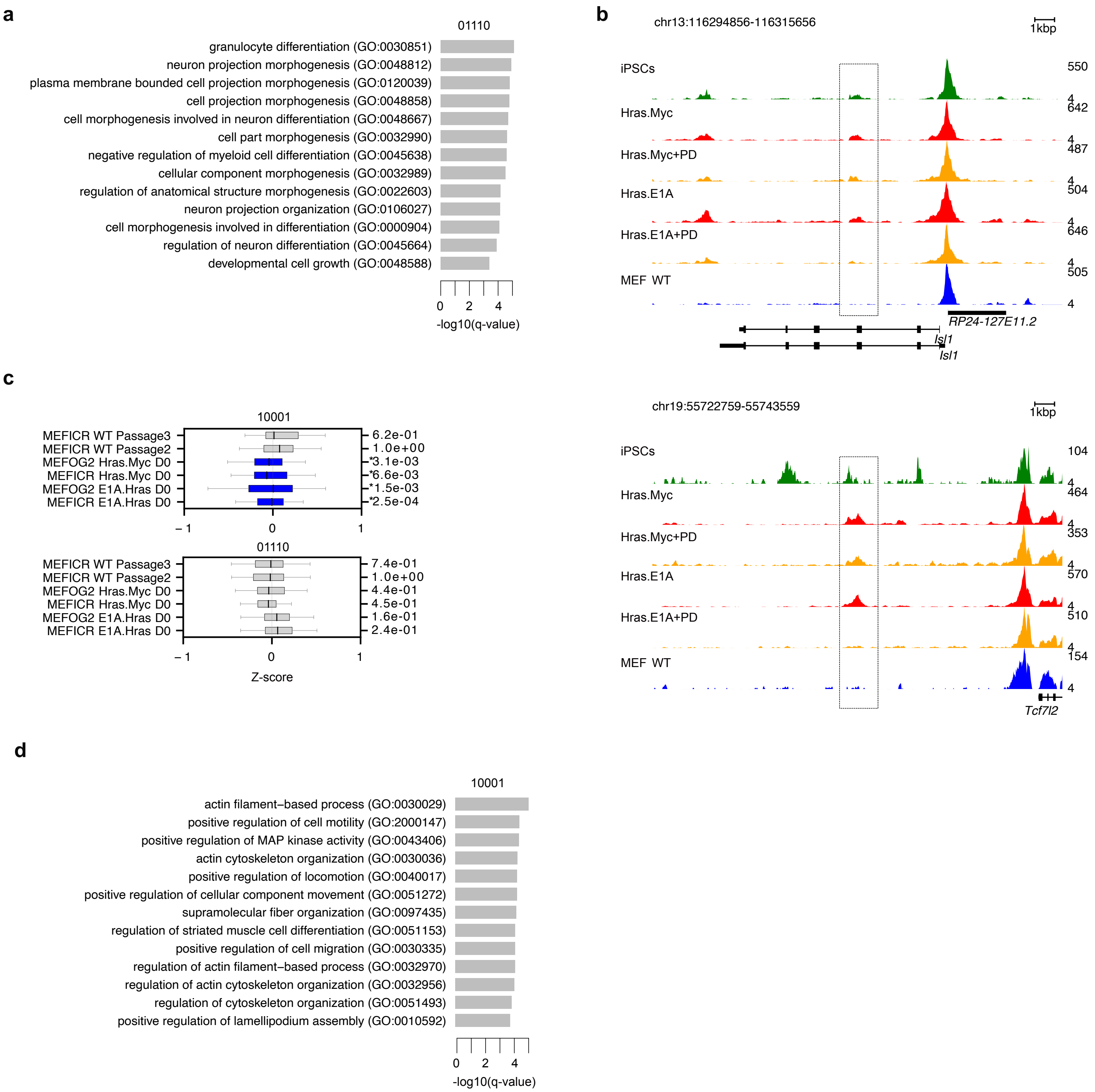
a
01110
granulocyte differentiation (GO:0030851)
neuron projection morphogenesis (GO:0048812)
plasma membrane bounded cell projection morphogenesis (GO:0120039)
cell projection morphogenesis (GO:0048858)
cell morphogenesis involved in neuron differentiation (GO:0048667)
cell part morphogenesis (GO:0032990)
negative regulation of myeloid cell differentiation (GO:0045638)
cellular component morphogenesis (GO:0032989)
regulation of anatomical structure morphogenesis (GO:0022603)
neuron projection organization (GO:0106027)
cell morphogenesis involved in differentiation (GO:0000904)
regulation of neuron differentiation (GO:0045664)
developmental cell growth (GO:0048588)
0 2 4
-log10(q-value)
b
chr13:116294856-116315656
1kbp
iPSCs
550
4
Hras.Myc
642
4
Hras.Myc+PD
487
4
Hras.E1A
504
4
Hras.E1A+PD
646
4
MEF WT
505
4
RP24-127E11.2
Isl1
Isl1
chr19:55722759-55743559
1kbp
iPSCs
104
4
Hras.Myc
464
4
Hras.Myc+PD
353
4
Hras.E1A
570
4
Hras.E1A+PD
510
4
MEF WT
154
4
Tcf7l2
c
10001
MEFICR WT Passage3
MEFICR WT Passage2
MEFOG2 Hras.Myc D0
MEFICR Hras.Myc D0
MEFOG2 E1A.Hras D0
MEFICR E1A.Hras D0
6.2e-01
1.0e+00
*3.1e-03
*6.6e-03
*1.5e-03
*2.5e-04
−1 0 1
01110
MEFICR WT Passage3
MEFICR WT Passage2
MEFOG2 Hras.Myc D0
MEFICR Hras.Myc D0
MEFOG2 E1A.Hras D0
MEFICR E1A.Hras D0
7.4e-01
1.0e+00
4.4e-01
4.5e-01
1.6e-01
2.4e-01
−1 0 1
Z-score
d
10001
actin filament−based process (GO:0030029)
positive regulation of cell motility (GO:2000147)
positive regulation of MAP kinase activity (GO:0043406)
actin cytoskeleton organization (GO:0030036)
positive regulation of locomotion (GO:0040017)
positive regulation of cellular component movement (GO:0051272)
supramolecular fiber organization (GO:0097435)
regulation of striated muscle cell differentiation (GO:0051153)
positive regulation of cell migration (GO:0030335)
regulation of actin filament−based process (GO:0032970)
regulation of actin cytoskeleton organization (GO:0032956)
regulation of cytoskeleton organization (GO:0051493)
positive regulation of lamellipodium assembly (GO:0010592)
0 2 4
-log10(q-value)

Supplementary Fig. 10. Chromatin at developmental enhancers is suppressed by MEK inhibition

- **a** GO analysis of the genes within 5000 bp of a chromatin locus in the 01110 group.
- **b** Genome views of two developmental genes, *Isl1* and *Tcf7l2*.
- **c** Boxplots showing the gene expression of the genes within 5000 bp of a chromatin locus in the 01110 or 10001 groups. Significance is from a two-sided Welch's t-test.
- **d** GO analysis of the genes within 5000 bp of a chromatin locus in the 10001 group.
